# Identity and validity of conserved B cell epitopes of filovirus glycoprotein: towards rapid diagnostic testing for Ebola and possibly Marburg virus disease

**DOI:** 10.1186/s12879-018-3409-x

**Published:** 2018-10-03

**Authors:** Peace Babirye, Carol Musubika, Samuel Kirimunda, Robert Downing, Julian J Lutwama, Edward K Mbidde, Jacqueline Weyer, Janusz T Paweska, Moses L Joloba, Misaki Wayengera

**Affiliations:** 1 Department of Immunology and Molecular Biology, School of Biomedical Sciences, College of Health Sciences, Makerere University, P o Box 7072, Kampala, Uganda; 2 Arbovirology and Filovirology Laboratories/Centers for Disease Control—CDC, Uganda Virus Research Institute (UVRI), Entebbe, Uganda; 3 Center for Emerging Zoonotic Diseases, National Institute for Communicable Diseases, Johanesburg, South Africa; 4 Unit of Genetics & Genomics & Department of Pathology, School of Biomedical Sciences, College of Health Sciences, Makerere University, P o Box 7072, Kampala, Uganda

**Keywords:** Ebolavirus, Marburgvirus, Viral hemorrhagic fevers, Rapid diagnostic tests, Glycoprotein

## Abstract

**Background:**

*Ebolavirus* and *Marburgvirus* are genera of the virus family *Filoviridae*. Filoviruses cause rare but fatal viral hemorrhagic fevers (VHFs) in remote villages of equatorial Africa with potential for regional and international spread. Point-of-care (POC) rapid diagnostic tests (RDTs) are critical for early epidemic detection, reponse and control. There are 2 RDTs for Zaire *ebolavirus* (EBOV), but not other *Ebolavirus* spp. or *Marburg marburgvirus* (MARV). We validate 3 conserved B cell epitopes of filovirus glycoprotein (GP) using ebola virus diseases (EVD) survivor samples, towards devising pan-filovirus RDTs.

**Methods:**

*In-silico* Immuno-informatics:- (a) multiple and basic local alignments of amino-acid sequences of filovirus (4 *Ebolavirus* spp. & MARV) Gp1, 2 and epitope prediction and conservation analyses within context of ClusterW, BLAST-P and the immune epitope database analysis resource (IEDB-AR); alongside (b) in-vitro enzyme immuno-assays (EIAs) for SUDV Gp1, 2 antigen and host-specific antibodies (IgM and IgG) among 94 gamma irradiated EVD survivor serum and 9 negative controls.

**Results:**

Linear B cell epitopes were present across the entire length of all Gp1, 2, most lying in the region between amino acids positioned 350 and 500. Three seperate epitopes 97/80_GAFFLYDRLAST, 39_YEAGEWAENCY and 500_CGLRQLANETTQALQLFLRATTELR (designated UG-Filo-Peptide− 1, 2 and 3 respectively) were conserved within all studied filovirus species Gp1, 2. Gp1, 2 host specific IgM levels were comparably low (av. ODs < 0.04 [95% CI: 0.02837 to 0.04033]) among the 9 negative controls and 57 survivor samples analyzed. Host specific IgG levels, *on the other hand,* were elevated (av. ODs > 1.7525 [95% CI: 0.3010 to 3.1352]) among the 92 survivor samples relative to the 9 negative controls (av. ODs < 0.2.321 [95% CI: -0.7596 to 0.5372]). Filovirus Gp1, 2 antigen was not detected [av. ODs < 0.20] within EVD survivor serum relative to recombinant protein positive controls [av. ODs = 0.50].

**Conclusions:**

These conserved B cell epitopes of filovirus Gp1, 2 and their derivative antibodies are promising for research and development of RDTs for EVD, with potential for extension to detect MVD.

**Electronic supplementary material:**

The online version of this article (10.1186/s12879-018-3409-x) contains supplementary material, which is available to authorized users.

## Background

*Ebolavirus* and *Marburgvirus* are genera of the virus family *Filoviridae*. Filoviruses are enveloped, non-segmented single-stranded RNA viruses of the order *Mononegavirales* [1]. Both genera have virion particles that are pleomorphic with a long and filamentous—essentially bacillary structure [1, 2] *.* Their virions comprise: a nucleocapsid (NC), a cross-striated helical capsid, an axial channel in the nucleocapsid, and a surrounding lipoprotein unit (LP) derived from the host cell. The lipoprotein envelope is insterspersed with glycoprotein (GP) spikes [2].

Two filoviruses cause rare but fatal viral hemmorhagic fever (VHFs) in remote villages of equatorial Africa, with potential for regional and international spread [1, 2]. A member of the genus *Marburgvirus* was first isolated in 1967 during outbreaks in Germany and Yugoslavia. These outbreaks were linked to infected monkeys imported from Uganda [2]. Members of the genus *Ebolavirus*, on the other hand, first emerged in 1976 as the causative agent of two simultaneous VHF outbreaks in southern Sudan and northern Zaire [1, 3]. Since then, species (*spp*) of the two genera have caused several outbreaks of VHFs, some designated public health emergencies of international concern (PHEIC) [3, 4]. Five species of the genus *Ebolavirus*, four of which are pathogenic to man (*Sudan ebolavirus-SUDV, Zaire ebolavirus (EBOV), Tai Forest ebolavirus-TAFV,* and *Bundibugyo ebolavirus-BDBV*). *Reston ebolavirus-RESTV* has only been linked to VHF-like illness among non-human primates (NHPs) [5]. On the contrarily, there is only one species of the genus *Marburgvirus* (denoted *Marburg marburgvirus* or simply *marburg virus*: MARV) with multiple genetic lineages [4, 6]. The high infectiousness and case-mortality rates (23–95%) associated with either VHFs warrants the designation of both filovirus genera as class A pathogens [4, 6, 7].

Though laboratory diagnosis of the two filoviruses is possible, the available technology platforms lack the user-friendliness for use at the point-of-care (POC). This because most remote villages of equatorial Africa where the index cases occur, lack the laboratory set-up needed to ran the current tests. Saijo M, et al. [8] have previously reviewed the laboratory diagnostic systems for ebola and marburg VHFs developed with recombinant proteins, including viral culture, antigen capture and host specific antibody response (IgM and IgG) assays. Elsewhere, pyro- and next-generation sequencing (NGS), and reverse transcriptase (RT) based PCR –ordinary or nested, have been described for filovirus diagnosis basing on nucleic acids amplification testing (NAATs). Between all methods, antigen-capture/host-antibody enzyme linked immunoabsorbent assays (ELISAs) and NAATS can theoretically safely be performed—after specimen sterilization, within laboratories with less than biosafety level –IV (BSL-4) containment. However, given the potential risks of transmission associated with laboratory mishandling, all suspected filovirus specimen must practically be handled within mimumum biosafety level-3 containment, and culturing of virus is restricted to BSL-4 facilities [8]. This picture underscores the need to develop biomarkers of acute and or late filovirus infection to mount on easy- to- use, cheap technology platforms that are suited for testing at POC [9–11]. Recent efforts have developed RDTs for Zaire *ebolaviru*s (EBOV) namely: the Corgenix ReEBOV® and OraSure Technlogies., Inc. OraQuik® EBOV rapid antigen test. As far as we are aware, both these RDTs were not designed with the multi-purpose of detecting other *Ebolavirus* species and or MARV [12, 13]. In addition, both target the EBOV VP40 antigen rather than GP.

Filovirus GP is used for virus cell targeting and entry by mediating receptor binding and membrane fusion [14–17]. GP comprises 2 subunits (GP1 and GP2) linked through a disulfide-bond generated after proteolytic cleavage of the GP precursor (Gp1, 2) by the cellular subtilisin-like protease furin [18, 19]. Surface GP is a trimeric type I transmembrane protein (tGP) that forms structural spikes on the exterior of infected cells and virions [19]. Due to differences in transcriptional editing, MARV only exhibits the transmembrane type of GP (tGP), while ebola virus also manifests a secretory form of GP (sGP). In contrast to *Ebolavirus* species that engage transcriptional editing to express the secretory form of GP (sGP), the GP gene of MARV is organized in a way that transcription results in a single sub-genomic RNA species used for the synthesis of the full-length envelope GP [20, 21]. Thus, MARV does not express the secretory form of the glycoprotein (sGP) that is synthesized from the edited mRNA during *Ebolavirus* species infection and secreted into the culture medium [21]. Expression of tGP by ebola virus is limited during virus replication, since most GP gene-specific mRNAs (80%) is directed towards synthesis of the secreted non-structural glycoprotein (sGP) [20]. In addition, significant amounts of tGP are shed from the surface of infected cells due to cleavage by the cellular metalloprotease tumour necrosis factor alpha-converting enzyme (TACE) [22]. *Ebolavirus* and *Marburgvirus* species’ GP1, 2 preproteins share 31% identity in amino acid sequences of the N- and C-terminal regions. Inferably, this similarity (and 69% variability) may be exploited for pan-filovirus detection and or delineation of the various species [21, 23, 24].

In light of the frequent VHF outbreaks caused by members of the genera *Ebolavirus* and *Marburgvirus* in Uganda, our group set out to identify conserved B cell epitopes of the filovirus GP1,2 preprotein that could be harnessed towards research and development (R & D) of a multi-purposed RDT for screening for all filoviruses (pan-filovirus). We report the validation of 3 conserved B cell epitopes of the filovirus glycoprotein (GP) using EVD survivor samples (SUDV spp). Note that seperate RDT versions detecting either GP antigen or host specific antibodies (IgM and IgG) are envisaged, and our report of only positive results for IgG, does not imply otherwise. Instead, the inability to capture GP antigen and its host-specific IgM in survivor samples is further validation of the accuracy of our targets since the pathogenesis of EVD repudiates their existence at the time the survivor sample were collected. Supplementary testing with MARV samples is, however, still required to experimentally affirm duo-usage.

## Methods

### Identification of conserved B cell epitopes of filovirus GP1, 2 pre-protein

- The primary amino acids sequences of the GP1, 2 preprotein for 4 *Ebolavirus* species [*Zaire ebolavirus* (strain Eckron-76)= > sp.|P87671|, *Tai Forest ebolavirus* (strain Cote d’Ivoire-94)= > sp.|Q66810|, *Sudan ebolavirus* (strain Maleo-79)= > sp.|Q66798| and *Reston ebolavirus* (strain Philippines-96)= > sp.|Q91DD8|] alongside one *Marburgvirus* species [*Marburg marburgvirus,* MARV (strain Angola/2005)= > sp.|Q1PD50|] were separately fed into the interfaces of the immune epitope database analysis resource (IEDB-AR) [25] and Bebipred [26]. Four biophysical profiles (beta-turn, surface accessibility, hydrophilicity and antigenicity) were evaluated alongside the hidden Markov BeBipred propensity as per user protocols [27].
- The same amino acids sequences were simultaneously fed into the user interface of the ClustalW software and conserved linear peptides derived according to the user protocols at default [28]. The derivative conserved epitopes were (i) queried against the National Center for Biotechnology Information (NCBI) human proteome, microbial proteome database, fungal proteome database, and protozoa proteome database and Conserved Domain Database (CDD) by BLAST-P [29, 30] and (ii) scanned against the 3-D crystal structure of Ebola GP in combination with antibodies from a human survivor (PDB entry 3CSY) using Prosite Scan [10, 31]. Details of the methodologies are available in reference [32].
- Avialibility of Software and Databases
  - The IEDB-AR resource used in this paper is available at the following url: http://tools.immuneepitope.org/main/
  - The linear B cell epitope prediction profiles used in this study are available at the following url: http://tools.immuneepitope.org/tools/bcell/iedb_input
  - The BepiPred software used in this analysis is available at the following url: http://www.cbs.dtu.dk/services/BepiPred
  - The Clustal W multiple alignment software and algorithms is available at the following url: www.ebi.ac.uk/clustalw/
  - The NCBI database of 2107 microbial proteomes used further above, alongside its BLAST-P tool are available at the following url: http://www.ncbi.nlm.nih.gov/sutils/genom_table.cgi
  - The reference proteome of the Human genome along with its BLAST tool are available at the following url: http://www.ncbi.nlm.nih.gov/blast/Blast.cgi
  - The Protscan software and algorithms is available at the following url: http://www.expasy.org/tools/scanprosite/
  - The PDB macromolecular structure database hosting the 3- dimensional structure of EBOV GP entry 3CSY is available at the following url: http://www.ebi.ac.uk/msd-srv/msdmotif/

### Validation of filovirus GP1, 2 antigen and host-specific antbody (IgM and IgG) detection

*Design:* Cross-Sectional Laboratory Study.

*Site:* Immunology laboratory, Department of Immunology and Molecular Biology, School of Biomedical Sciences, College of Health Sciences, Makerere University Kampala, Uganda.

*Samples and participants:* 94 Ebola Virus Disease (EVD) gamma-irradiated survivor serum samples (collected during the 2000 Outbreak of *SUDV* in Gulu and Masindi, and advanced to us courtesy of Uganda Virus Research Institute—UVRI and Centers for Disease Control—CDC, Entebbe, UG) alongside 9 EVD negative controls. All these samples were broadly consented for future use, and a waiver clearance was obtained from the Institutional Review and Ethics Committee (IREC) to re-use them in this work. No MARV samples were included.

*Materials and Reagents*: Synthetic analogues of the filovirus GP1, 2 peptide epitopes 1, 2 and 3 (denoted UG-Filo-Peptide 1, 2 and 3 respectively, GeneCUST, Luxemburg), New Zealand Rabbit derived anti-UG-Filo-Peptide 1 and anti-UG-Filo-Peptide 3 polyclonal antibodies (denoted PAbs- A005345 and A005346 respectively), plain ELISA plates (flat bottom, Nunc), Bovine Serum Albumin (BSA, In-vitrogen, USA), recombinant EBOV GP1, 2, goat anti-human IgM and IgG (HRP labeled, Bio-Rad, France), Phosphate Buffered Saline (PBS), and the enzymatic substrate tetramethylbenzidine (TMB).

*Interventions* (a) **Synthetic Epitopes:** Amino acid sequences of the epitopes UG-Filo-Peptide 1, 2 and 3 were loaned to GeneCUST, Luxemburg, for biochemical manufacture of synthetic analogues of the same. (b) **Cloning and Expression of recombinant EBOV GP1, 2 Protein:** Amino acid sequences of *Zaire ebolavirus (*EBOV*)* sp.|Q66798| were loaned to GenSCRIPT, HGK for sub-cloning and expression of the recombinant protein in HEK293-6E cell-lines. (c) **Filovirus GP1, 2 host specific IgM or IgG antibodies detection EIA:** For detection of Filovirus GP1, 2 host specific IgM and IgG humoral responses in serum of 92 (of 94) EVD survivor serum, we (i) dissolved 1μg (conc: 1 mg/ml) of individual synthetic peptide by adding 100 μl of freshly prepared phosphate buffered saline (PBS was prepared by dissolving ¼ of a 250 mg tablet in 50 ml PCR grade water). (ii) 100 μl (0.001 ng) of individual synthetic peptide (UG-Filo-Peptide-01 & UG-Filo-Peptide-02) was then pipetted into each of the wells of a sterile 96-well microtiter plate (Nunc) and the plate incubated overnight. (iii) The plated wells were then blocked once the following day using 5% BSA in PBS and incubated at 37 °C for 30 mins, after which excess solution was discarded and plate left to dry. (iv) 100μls of PBS was added to each assigned wells, followed by addition of 10 μl (1:100 dilution) of samples into the respective wells; after which the plate was shaken at 15HZ for 16 s, and then incubated for 1 h at 37 °C. Blank wells were also made, by adding only PBS rather than sample. The wells-in-use were then washed with PBS three times using an automated plate-washer. (v) 100μls of either goat anti-human IgM or IgG horseradish peroxidase conjugate was added, and the plates incubated at 37 °C for another 1 h. During this incubation, the enzyme substrate was prepared by adding 1 volume of substrate (TMB) to 1 volume of diluent (hydrogen peroxide) in volumes enough for all the wells in use. (vi) 200 μl of freshly prepared substrate was added to each well (purple-bluish color developed in all except A-BX1 blank wells). (vii) The reaction was stopped by adding 100 μl of dilute (1 mol/L) H_2_SO_4_. The intensity of the reaction in each well was hence after determined by reading the plate at an optical density (OD) of 450 nm using a single filter of an automated ELISA plate reader (PR 3100, Bio-Rad). (d) **Filovirus GP1,2 antigen EIA**. For detection of *Filovirus* GP1, 2 antigen (Ag) among serum of the 92 EVD survivors (i) dissolved 1uL of serum was dissolved in 1000 μl or 1 ml of freshly prepared phosphate buffered saline. (ii) 100 μl of resultant serum-diluent was then pipetted into each of the wells of a sterile 96-well microtiter plate (Nunc) and the plate incubated overnight. (iii) The plated wells were then blocked once the following day using 5% BSA in PBS and incubated at 37 °C for 30 mins, after which excess solution was discarded and plate left to dry. Blank wells were also made, by adding only PBS rather than sample. The wells-in-use were then washed with PBS three times using an automated plate-washer. (iv) 100uL of either PAb-A005345 or -A005346 (1 mg/ml reconstituted in 5000 of PBS) rabbit derived was added and plates incubated at 37 °C for 30 mins, after which excess solution was discarded and plate left to dry. The wells-in-use were then washed with PBS three times using an automated plate-washer. (v) 100μls of goat anti-rabbit IgG horse-raddish peroxidase conjugate was added, and the plates incubated at 37 °C for another 1 h. During this incubation, the enzyme substrate was prepared by adding 1 volume of tetramethylbenzidine substrate (TMB) to 1 volume of diluent (hydrogen peroxide) in volumes enough for all the wells in use. (vi) 200 μl of freshly prepared substrate was added to each well (purple-bluish color developed in all except A-BX1 blank wells). (vii) The reaction was stopped by adding 100 μl of dilute (1 mol/L) H_2_SO_4_. The intensity of the reaction in each well was hence after determined by reading the plate at an optical density (OD) of 450 nm using a single filter of an automated ELISA plate reader (PR 3100, Bio-Rad).

*Measured Variables:* Levels of host specific IgM and IgG antibodies as swell as filovirus GP1, 2 Ag in study serum or blanks were qualitatively detected as a function of the OD of each well. Statistically, measures of best-fit, standard error, 95% confidence interval and goodness of fit were obtained.

*Treatment of Results:* Raw data was cleaned by subtracting ODs of blanks from those of test wells. The issuing adjusted ODs were either run as triplicates in GraphPad® (IgM and IgG) or averaged across the triplicates runs for each test (Excel). Resultant average adjusted ODs were analyzed by both PRISM® software, and Excel®. Graphs were also drawn by GraphPad®. For each OD read (essentially done in duplicate), a 95% confidence interval (CI) was computed, alongside the slopes and *P*-values. Excel sheets were used for correction of average sample OD readings by subtracting OD reading of the blank wells.

## Results

### Identity of conserved B cell epitopes of filovirus GP1,2 pre-protein

**Fig. 1:**
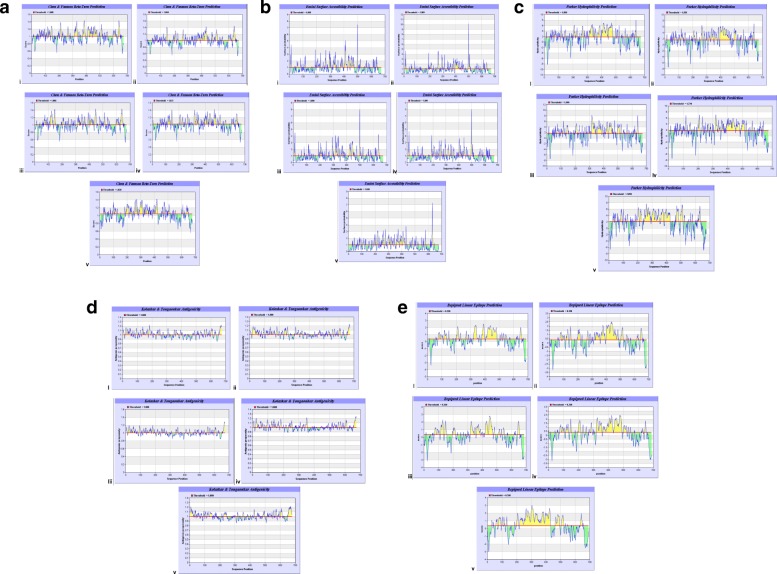
Distribution of five biophyical profiles along GP1, 2 preprotein of the 5 study ebola virus/MARV species. This figure is a graphic representation of the distribution of the five biophysical profiles along GP1, 2 preprotein of the 4 study *Ebolavirus* spp. and MARV: Z*aire ebolavirus*, *Tai forest ebolavirus*, S*udan ebolavirus*, R*eston ebolavirus*, and *Marburg marburgvirus.* Plates A-to E represent the biophysical profiles beta-turn, surface accessibility, hydrophilicity, antigenicity and Bepipred. Slides I to V within each of plate A-through-E represent profiles across the 4 *Ebolavirus* spp. and MARV, respectively

**Fig. 2:**
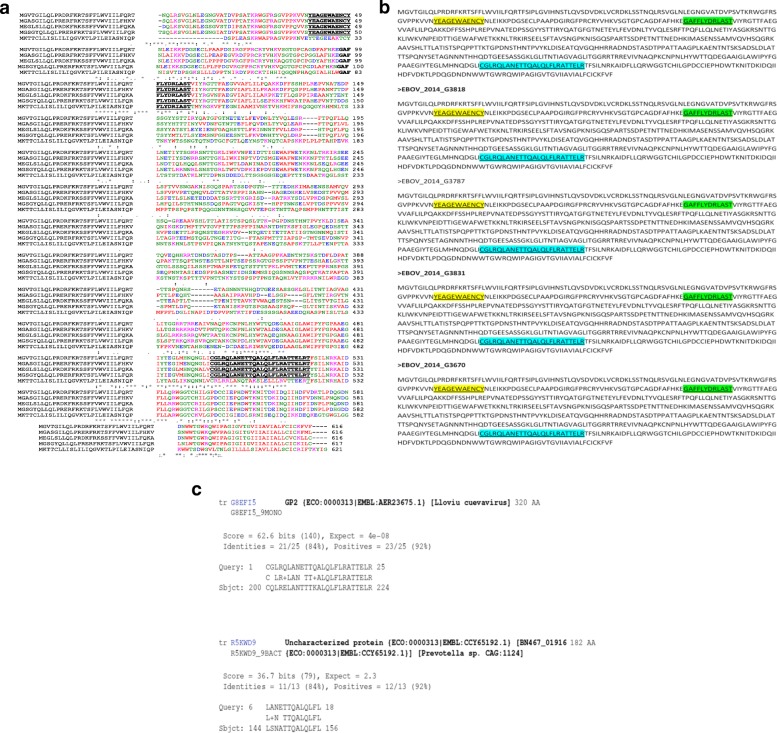
Conserved *Filovirus* GP1, 2 Pre-protein Epitopes by Multiple and Basic Local Sequence Alignments. This figure shows the 3 highly conserved Filovirus GP1, 2 Pre-protein epitopes as determined by ClusatlW and BLAST-P. Plate A shows the distribution of the epitopes across the study 4 *Ebolavirus* spp. and MARV GP1, 2 pre-proteins. Plate B reveals the two sources of cross-reactivity with UG-Filo-Peptide 3 predicted by the SIB-BLAST-P tool. Plate C details the distribution of the 3 epitopes within the GP1, 2 of the Zaire ebolavirus (EBOV) strain associated with the 2013–2016 EVD outbreak in West Africa

**Fig. 3:**
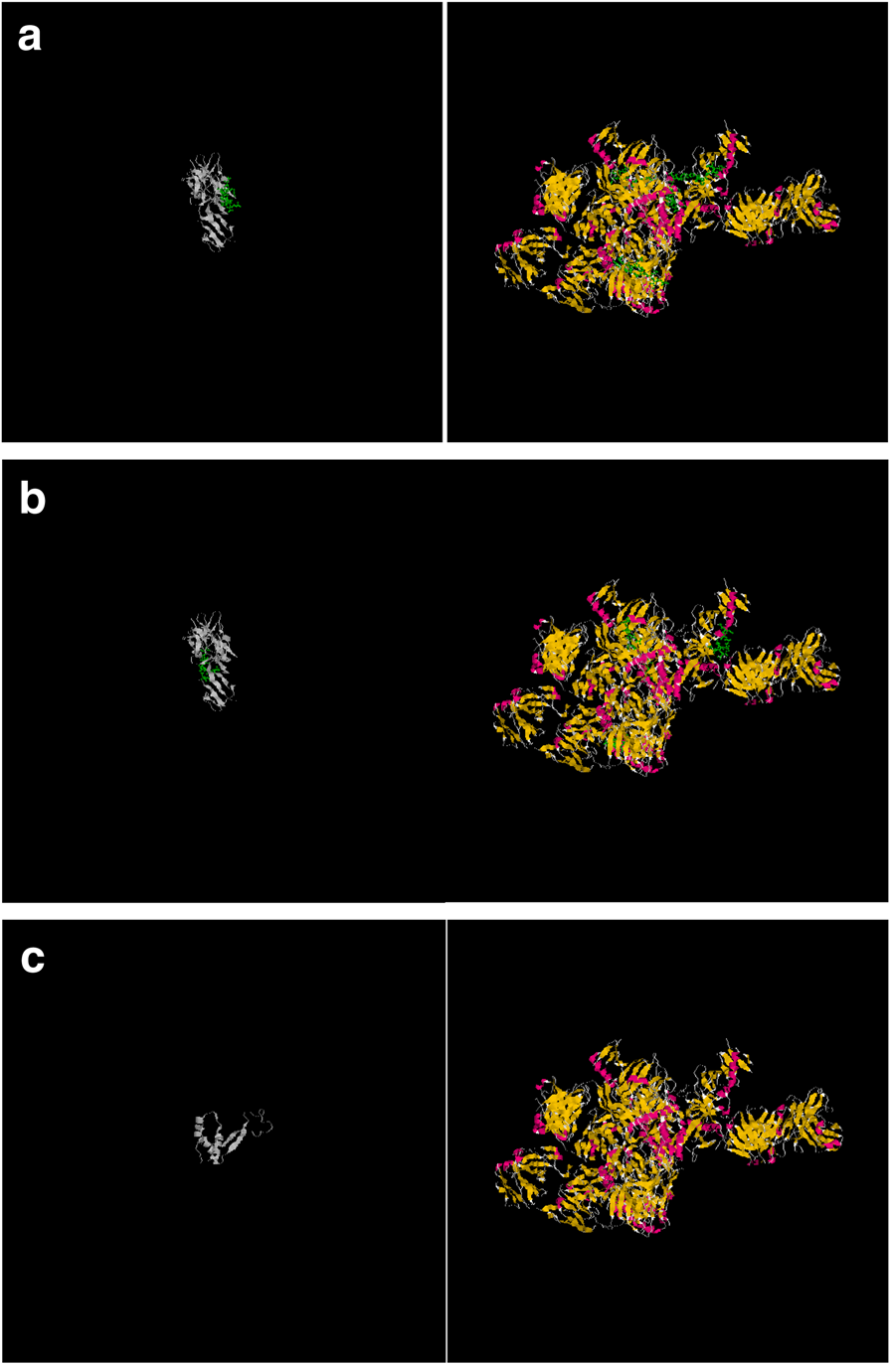
Localization of of the 3 conserved epitopes on the 3-D crystal structure of EBOV GP1, 2, pre-protein. This figure shows localization of of the 3 conserved epitopes (UG-Filo-Peptide 1, 2 and 3) on the 3-D crystal structure of EBOV GP1, 2 bound to an antibody from a human survivor, PDB entry 3CSY . Plates A, B and C show coordinates of the UG-Filo_peptide 1, 2 and 3 respectively

The distribution of B cell epitopes within the filovirus GP1,2 pre-proteins of 4 ebola virus and 1 MARV species analyzed using the IEDB-AR was even across the length of all species GP (see Fig. 1: Plates A, B, C, D and E). Plates A-to E represent the biophysical profiles beta-turn, surface accessibility, hydrophilicity, antigenicity and Bepipred. Slides I to V within each of plate A-through-E represent profiles for the 4 *Ebolavirus* spp. and MARV, respectively. The threshold values for the 4 biophysical profiles and Bepipred against the 4 *Ebolavirus* spp./MARV analyzed are shown in Table 1. Note that, other than the profile of antigenicity (see Fig. 1: Plate D) [25], all other profiles (see Fig. 1a, b, c and e) predicted that the best B cell epitopes of all *Ebolavirus* spp./MARV GP1,2 pre-proteins analyzed correspond to amino acid residues localized between positions N_350 and C_500. Considering the average length of the 4 *Ebola virus/MARV* spp. GP1, 2 preprotein, this region comprises of middle placed residues, which have previously also been shown to exhibit the highest level of variability across filovirus GP1, 2 [22]. The biophysical profile of hydrophilicity—used as the best propensity for our Bepipred analyses in section (ii) below, offered highest prediction of B-cell epitopes in this region (see Fig. 1: Plate E). These data demonstrate that the entire length of the 5 GP1,2 preproteins studied is interspersed with linear B cell epitopes that are usable for the overall goal developing filovirus diagnostics [32]. A previous study of the biophysical profiles of *Ebolavirus* spp. and MARV GP1,2 preproteins authored by our group found them to have predictably suitable extinction coefficients, instability index, and in-vivo half-lifes in mammalian cells, despite obvious proteomic and atomic differences [33]. Selection of strain-specific diagnostic linear peptides from within the single most highly epitopic region of both *Ebolavirus* spp. and MARV GP1, 2 was achieved using Bepipred--a combination of the hidden Markov model and best biophysical propensity (hydrophilicity) [27]. Once again, the highest prevalence of species specific B cell epitopes of the 4 *Ebolavirus* spp. and MARV GP1,2 was found to reside with amino acid residues in the region between position N_ 350 and C_500 by Bepipred (for illustration, see Fig. 1: Plate E). A list of the longest peptides derived is shown in Table 2. Identification of B cell epitopes common to all filovirus species studied was done using a combination of multiple sequence alignments of the 4 *Ebolavirus* spp. and MARV species’ GP1, 2 pre-protein in conjunction with the epitope predictions described above. The epitope 97/80_GAFFLYDRLAST (ssGP) was conserved across all filovirus GP1, 2, while 39_YEAGEWAENCY (GP) & 500_CGLRQLANETTQALQLFLRATTELR (GP2) were unique to only GP1, 2 of the 4 *Ebolavirus* species [32]. Details of the multiple alignments of the sequences of GP1, 2 preproteins for the 4 *Ebolavirus* spp. *and* MARV studied are shown in Fig. 2: Plate A. In order to computationally evalaute the specificity of these short peptides, we conducted protein basic local sequence alignment (BLAST-P) across proteome-wide databases (PwDB) of (a) homo-*sapiens*, (b) over 874 microbes and (b) a 27 protozoa PwDB including pathogens like *plasmodia*, *trypanosoma* and *leishmania* (c) the HIV sequence database, and (d) a 20 organismal fungal PwDB including *Aspergillus, Candida or Cryptococcus.* No matches were found for epitopes UG-Filo-Peptides 1 and 2. However, potential for cross reactivity of UG-Filo-Petide 3 was found with the GP2 of the C*uevavirus Lliovu* (LLOV) and an uncharacterized protein of the bacteria Prevotella *sp. CAG.1124* (see Fig. 2: Plate B), which might explain high IgM and IgG detection by this peptide (see Section B). All 3 epitopes were found to be present in the GP1, 2 of Zaire e*bolavirus* (EBOV) responsible for the 2014-to-2016 West African EVD outbreak (see Fig. 2: Plate C) [34]. Lastly, a prosite scan of these peptides against the 3-D crystal structure of EBOV GP1, 2 bound to an antibody from a human survivor, revealed the hits shown in Fig. 3: Plates A, B and C) [10]. Details of these data are described extensively in PCT filed at the World Intellectual Organization (WIPO) # PCT/IB2014/066251 [32].

**Table 1:** Showing the species and mean threshold score values for all biophysical profiles and Bepipred. This table depicts the mean threshold scores of 4 biophysical profiles and Bepipred in the IEDB-AR across the filovirus species

Biophysical Profile	Threshold score (Z*aire;* Tai forest; S*udan;* R*eston ebolaviruses; L.victoria marburgvirus*)	Mean Threshold score
Beta turn	1.008; 1.004; 1.005;1.022;1.039	1.016
Surface accessibility	1.000; 1.000; 1.000; 1.000; 1.000	1.000
Hydrophilicity	1.809; 1.569; 1.609; 1.720; 2.099	1.761
Antigenicity	1.000;1.000; 1.000; 1.000; 1.000	1.000
Bipred	0.350; 0.350; 0.350; 0.350; 0.350	0.350

**Table 2:** Showing the longest subtype specific linear B cell epitopes predicted by Bepipred**.** This table lists some of longest subtype specific linear B cell epitopes predicted by Bepipred

*EBOV/MBGV spp*	Peptide	Start position	End Position	Length
*Zaire, EBOV*	HHRRTDNDSTASDTPSATTAAGPPKAENTNTSKSTDFLDPATTTSPQNHSETAGNNNTHHQDTGEESASSGKLGLITN	407	484	78
*Tai Forest, TAFV*	DHSTTQPAKTTSQPTNSTESTTLNPTSEPSSRGTGPSSPTVPNTTESHAELGKTTPTTLPEQHTAASAIPRAVHPDELSGPGFLTNTIRGV	399	489	91
*Sudan, SUDV*	TMAPSPETQTSTTYTPKLPVMTTEEPTTPPRNSPGSTTEAPTLTTPENITTAVKTVWA	416	483	58
*Reston, RESTV*	STQGLTNGETITGFTANPMTTTIAPSPTMTSEVDNNVPSEQPNNTASIEDSPPSASNETIDHSEMNSIQG	364	433	70
*Marburg, MARV*	STKNQTCAPSKKPLPLPTAHPEVKLTSTSTDATKLNTTDPNSDDEDLTTSGSGSGEQEPYTTSDAATKQGLSSTMPPTPSPQPSTPQQGGNNTNHSQGVVTEPGKTNTTAQPSMPPHNTTTISTNNTSKHNLSTPSVPIQNATNYNTQSTAPENEQTSAPSKTTLLPTENPTTAKSTNSTKSPTTTVPNTTNKYSTSPSPTPNSTAQHLVYFR	220	432	213

### Validation of *filovirus* GP1, 2 antigen and host-specific antbody (IgM and IgG) detection

#### Synthetic epitopes

**Fig. 4:**
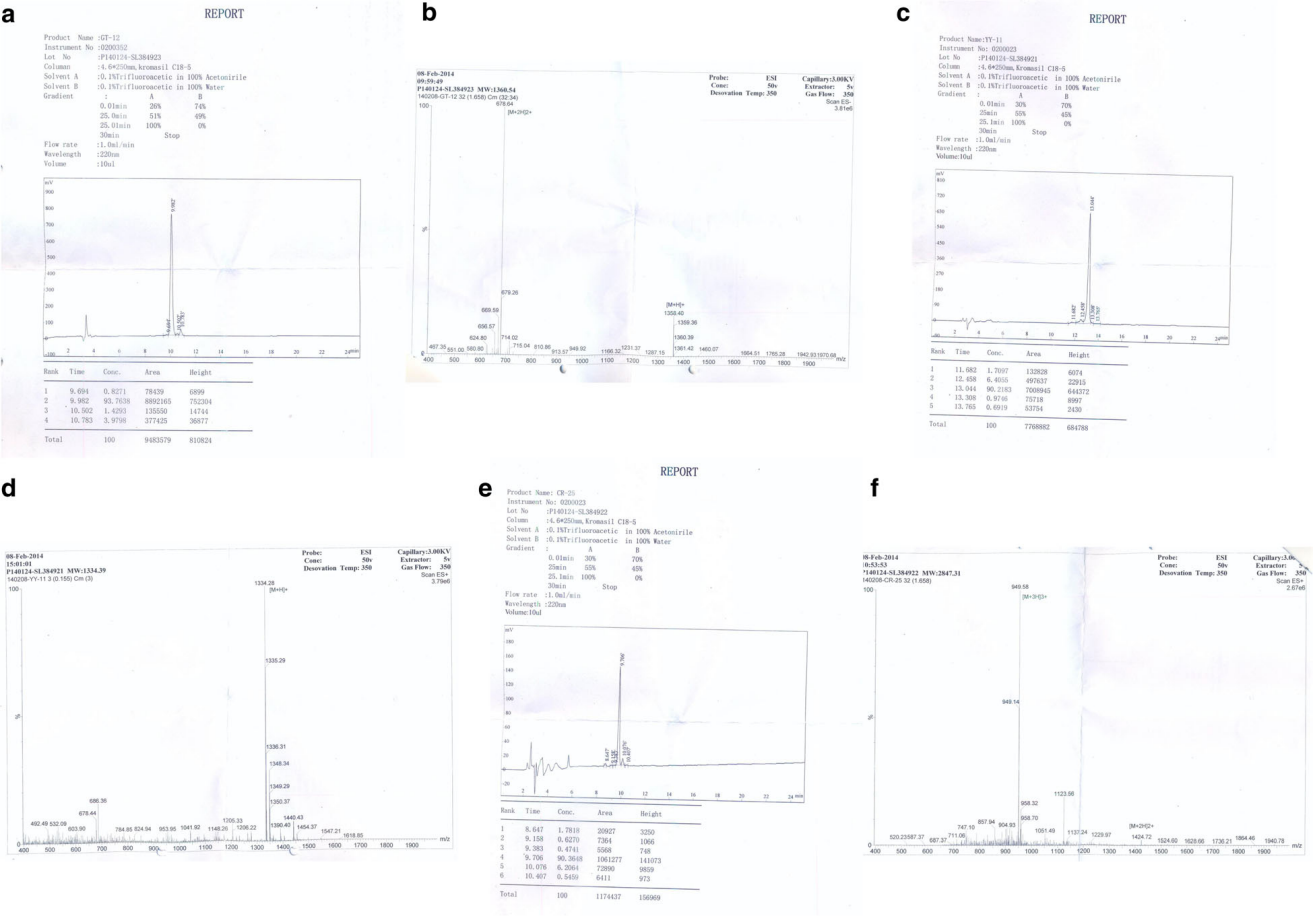
Mass-Spectrometry and high performance liquid chromatography of synthetic analogues of the 3 Conserved Epitopes of filovirus GP1, 2 pre-protein. This figure shows results of mass-spectrometry(MS) and high performance liquid chromatography (HPLC) of the synthetic analogues of the 3 highly conserved epitopes of filovirus GP1, 2 pre-protein. Plates A, C and E show MS, while Plates B, D and F show HPLC results

Synthetic analogues of the epitopes UG-Filo-Peptide 1, 2 and 3 were biochemically manufactured by GeneCUST., Luxemburg. Mass Spectrometry-MS and High Performance Liquid Chromatography-HPLC analysis results of purity for each are shown in Fig. 4: Plates A, B, C, D, E and F. Each epitope was supplied at a concentration of 1 mg/ml and 99% purity. KH-conjugated UG-Filo-Peptide 1 and 3 were used as immunogens among New Zealand Rabbits to generate the polyclonal antibodies (PAb-A005345 and PAb -A005346, respectively: see Additional file 1: S1).

#### Cloning and expression of recombinant EBOV GP1, 2 protein

**Fig. 5:**
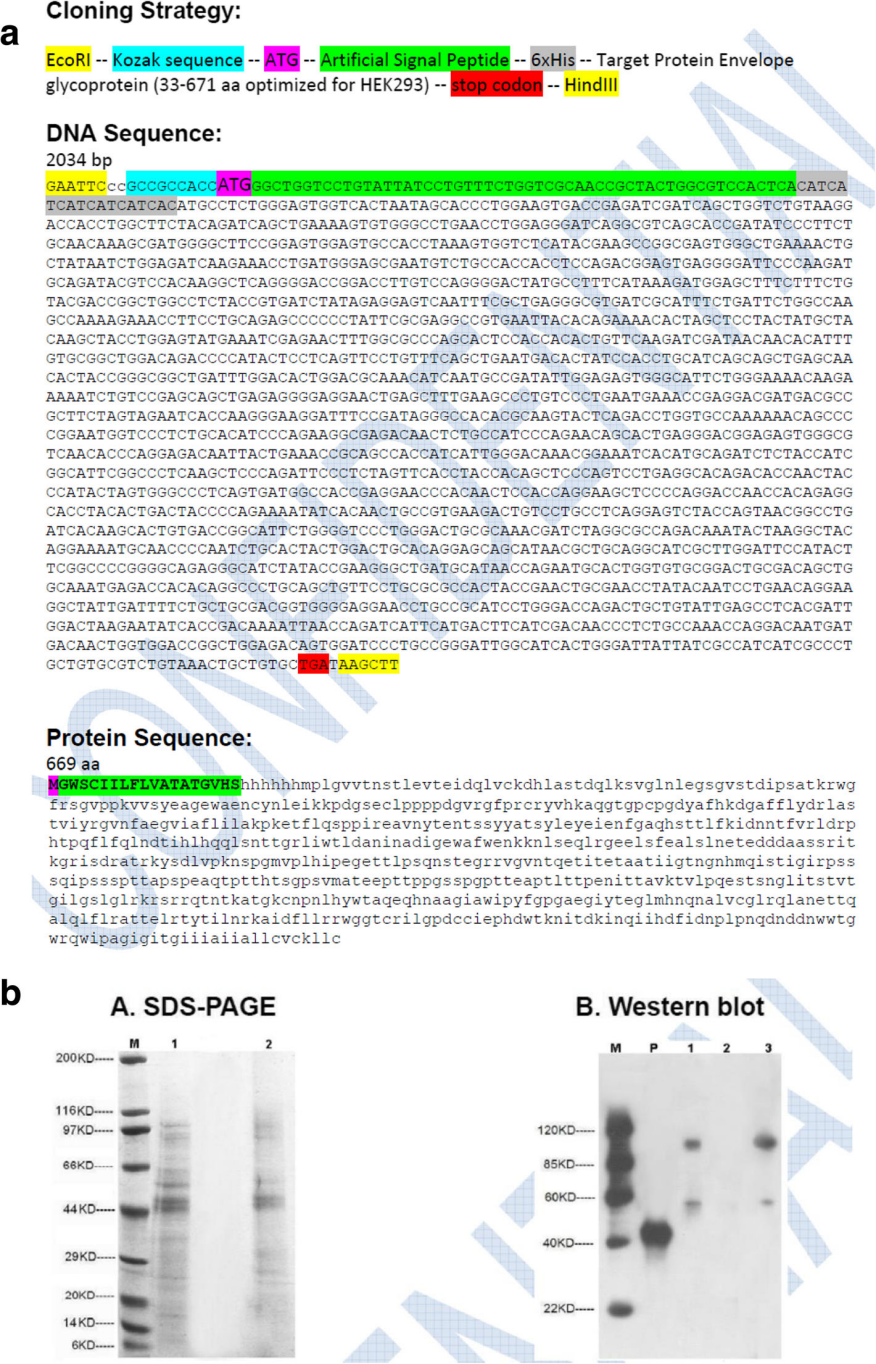
Coding DNA and SDS-PAGE analysis of recombinant EBOV GP1, 2 Pre-protein cloned and expressed in HEK293-6E mammalian cell-lines. This figure offers the coding (c) DNA and sodium dodecyl sulfate(SDS)-poly-acrylamide gel electrophoresis (PAGE) of recombinant EBOV GP1, 2 pre-protein cloned and expressed in HEK293-6E mammalian cell-lines. Plate A shows the cDNA while Plate B shows the SDS-PAGE analytes

Coding DNA of Zaire *ebolavirus* GP1, 2 sp.|Q66798| (see Fig. 4a) was sub-cloned into HEK293-6E cell-lines using electro-chemical poration (see Fig. 5a).Expressed protein was purified by Coomassie Blue-stained sodium dodecyl sulfate (SDS) gel electrophoresis (see Fig. 5b). The resultant purified rGP1, 2 was supplied at concentration of 0.2 mg/ml and purity of about 50%. For details, see Additional file 2: S2.

#### IgM detection EIA

**Fig. 6:**
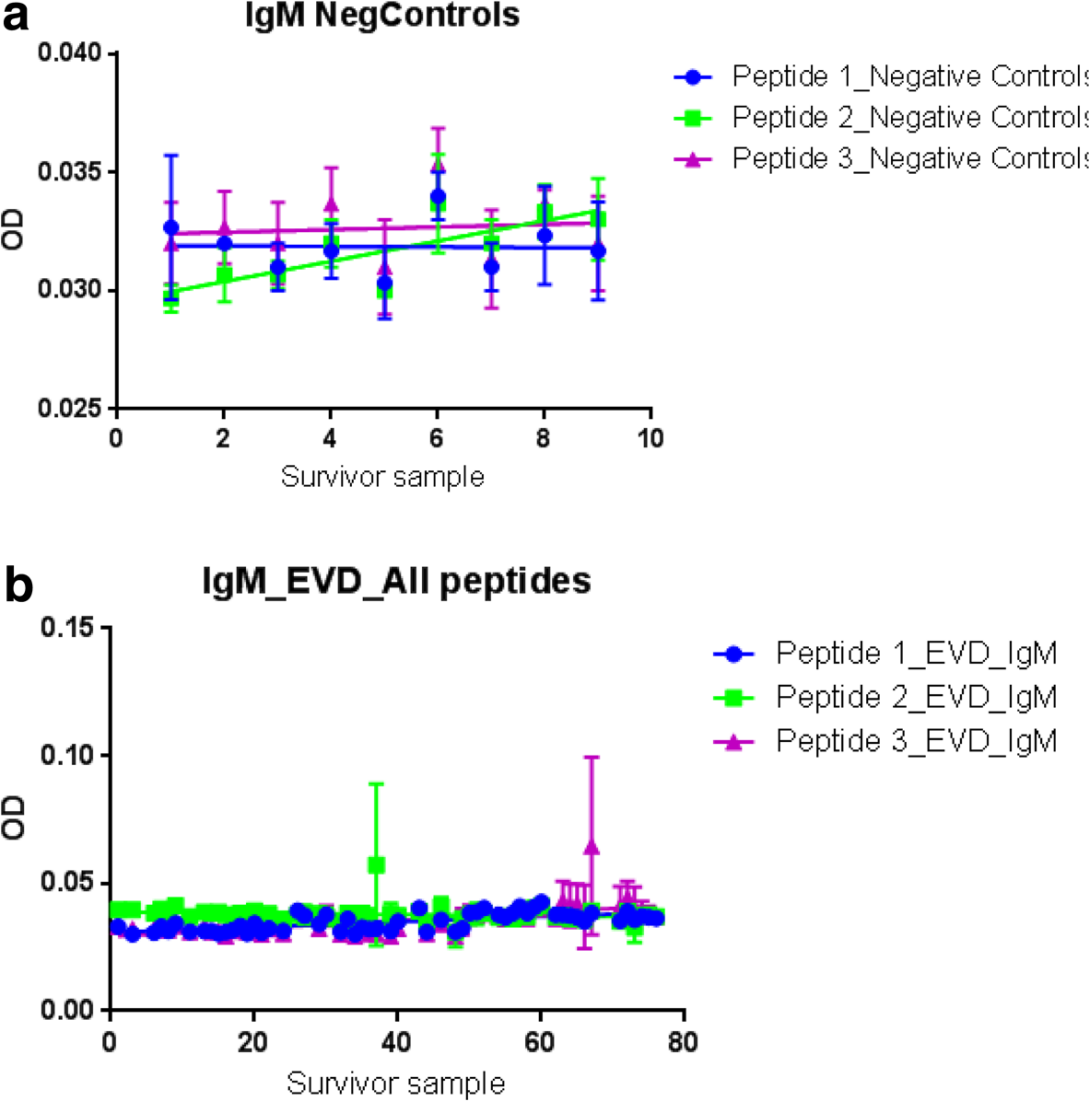
Host specific IgM levels among the negative controls and EVD survivor serum samples. This figure shows IgM levels among the negative controls and survivor serum samples. Plate A shows IgM levels among 27 runs of the 9 negative controls. Plate B shows 171 runs of 57 survivor serum samples. Note that IgM levels were generally low or absent among both negative controls and survivor serum samples

Filovirus Gp1, 2 host specific IgM levels were equally low (ODs < 0.04; 95% CI: 0.02837 to 0.04033) among 9 negative controls (see Table 3 and Fig. 6 Plate A) and 57 survivor samples analyzed (see Table 4 and Fig. 6 Plate B). Because all tests were run in triplicates, the total number of items analyzed was 27 for negative controls relative to 171 for survivor samples. Specifically, among the 9 negative controls, the 3 epitopes UG-Filo-Peptide 1, 2 and 3 detected IgM at ODs of 0.03191 (95% CI: 0.03039 to 0.03343), 0.02953 (95% CI: 0.02837 to 0.03069) and 0.03235 (95% CI: 0.03066 to 0.03404) respectively (Table 3). In concordance, the same epitopes UG-Filo-Peptide 1, 2 and 3 detected IgM at ODs of 0.03076 (95% CI: 0.02989 to 0.03163), 0.03876 (95% CI: 0.03720 to 0.04033) and 0.02914 (95% CI: 0.02710 to 0.03118) among the 57 survivor samples analyzed (Table 4). This picture is consistent with the immunopathogenesis of EVD—wherein IgM appears between 2 and 9 days after symptom onset, and disappears between 30 and 168 days after onset [34–38].

**Table 3:** *Filovirus* Glycoprotein (GP 1, 2) host specific IgM among Negative Controls. This table portrays host specific IgM levels among 9 Negative Controls

	Host Specific IgM		
	UG-Filo-Peptide 1	UG-Filo-Peptide 2	UG-Filo-Peptide 3
Best-fit values			
• YIntercept	0.03191	0.02953	0.03235
• Slope	-1.111e-005	0.0004278	5.556e-005
Std. Error			
• YIntercept	0.0007379	0.0005631	0.0008208
• Slope	0.0001311	0.0001001	0.0001459
95% Confidence Intervals			
• YIntercept	0.03039 to 0.03343	0.02837 to 0.03069	0.03066 to 0.03404
• Slope	−0.0002812 to 0.0002590	0.0002217 to 0.0006339	−0.0002449 to 0.0003560
Goodness of Fit			
• Degrees of Freedom	25	25	25
• R square	0.0002871	0.4223	0.005769
• Absolute Sum of Squares	7.739e-005	4.506e-005	9.574e-005
• Sy.x	0.001759	0.001343	0.001957
Number of points			
• Analyzed	27	27	27

#### IgG detection EIA

**Fig. 7:**
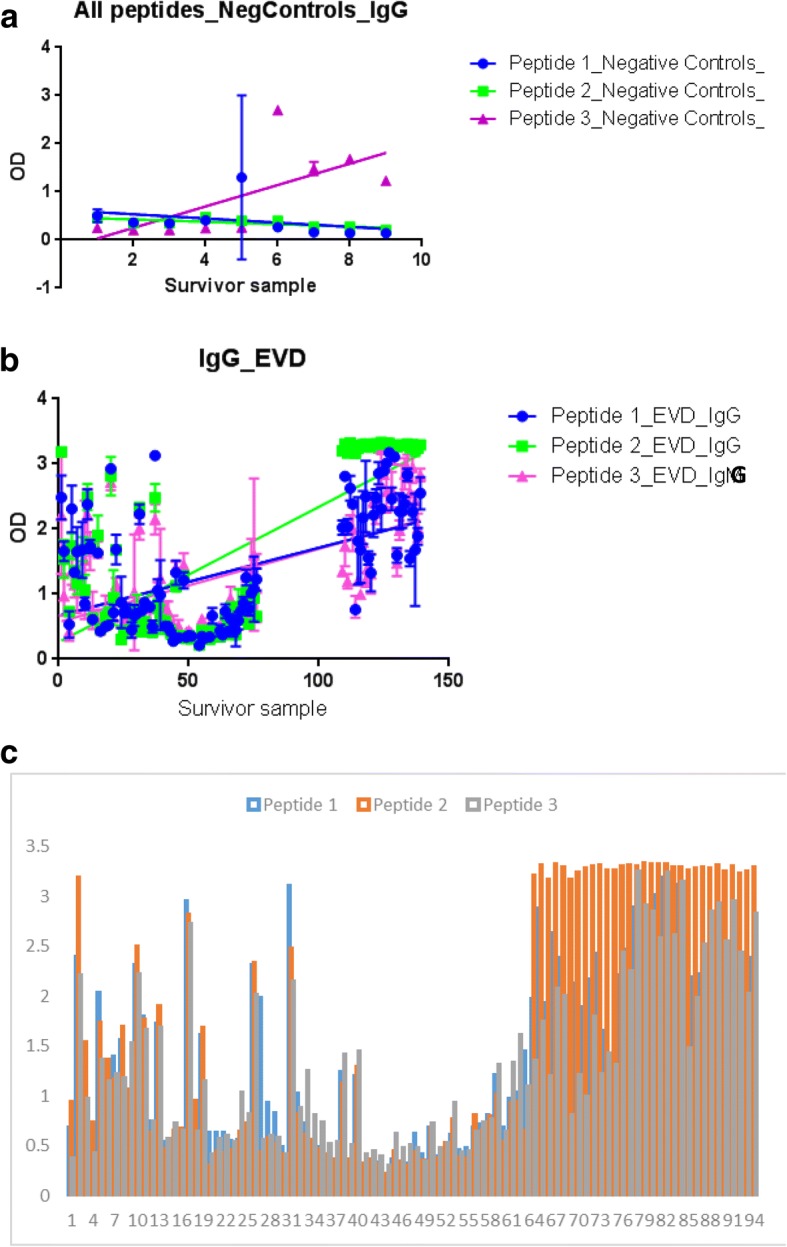
Host specific IgG levels among the negative controls and survivor serum samples. This figure depicts host specific IgG levels among the negative controls and survivor serum samples. Plate A shows IgG levels among 27 runs of the 9 negative controls. The two outliers hits for IgM within negative controls picked by UG-peptide 3 (Fig. 7a) are more likely to be due to either cross reactivity with another pathogen (see Fig. 2, plate B), or higher affinity, rather than the possibility that these were infected samples since they were drawn from a non-VHF endemic setting. Plate B shows 276 runs of 92 survivor serum samples. Note that IgG levels were elevated among survivor serum samples relative to negative controls. Plate C shows results of the differential ability of the 3 epitopes to capture host specific IgG among 282 runs of 94 survivor serum samples. Note the lower IgG titres detected by peptide 1 or 2 relative to peptide 3. These variations might be due to differences in peptide affinity to- and thereby sensitivity for- detecting host specific IgG

Host specific IgG levels, *on the other hand,* were elevated (av. ODs > 1.7525 [95% CI: 0.3010 to 3.1352]) among the 92 survivor serum samples relative to 9 negative controls (av. ODs < 0.2.321 [95% CI: -0.7596 to 0.5372]). Note that, because tests were ran in triplicates, the total number of analyzed survivor entries were 272 relative to 27 negative controls. Specifically, epitopes UG-Filo-Peptide 1, 2 and 3 respectively detected low IgG levels at ODs of 0.4165 (95% CI: 0.1125 to 0.7205), 0.4743 (95% CI: 0.4115 to 0.5372) and − 0.1944 (95% CI: -0.7596 to 0.3708) among the 9 negative controls (Table 5 and Fig. 7 Plate A). The three outliers hits for IgG within negative controls picked by UG-peptide 3 (Fig. 7a) are more likely to be due to cross reactivity with IgG responses to another pathogen (see Fig. 2, plate B, probably Provetella *spp*.), although higher affinity between antibody and this peptide is possible. It is therefore unlikely that these were *Ebolavirus* spp. (or even Lloviu *spp*) infected samples particularly since they were drawn from a non-VHF endemic setting. On the contrary, high levels of host specific IgG were detected among 92 analyzed survivor samples by the 3 epitopes UG-Filo-Peptide 1, 2 and 3 i.e. 1.7181 (95% CI: 0.3010 to 3.1352), 1.8197 (95% CI: 0.3268 to 3.3125) and 1.7197 (95% CI: 0.4145 to 3.0248) respectively (Table 6 and Fig. 7 Plate B). Once again, these data are generally consistent with prior studies that found that IgG antibody appears between days 6 and 18 after symptom onset and persists for life [34–38]. IgG is therefore expected to be abundant across the survivor samples. A separate EIA for host specific IgG ran across 94 survivor samples with the aim of identifying the best performing epitope, revealed that UG-peptide 3 had relatively higher specificity relative to the other two peptides across the statistical parameters used (see Table 7 and Fig. 7 Plate C). Details are available in Additional file 3: S3.

**Table 4:** *Filovirus* Glycoprotein (GP 1, 2) host specific IgM among Gamma Irradiated Ebola Survivor Samples. This table shows host specific IgM levels among 57 gamma irradiated EVD survivor samples

	Host Specific IgM		
	UG-Filo-Peptide 1	UG-Filo-Peptide 2	UG-Filo-Peptide 3
Best-fit values			
• YIntercept	0.03076	0.03876	0.02914
• Slope	0.0001031	−2.404e-005	0.0001580
Std. Error			
• YIntercept	0.0004409	0.0007923	0.001033
• Slope	9.769e-006	1.756e-005	2.302e-005
95% Confidence Intervals			
• YIntercept	0.02989 to 0.03163	0.03720 to 0.04033	0.02710 to 0.03118
• Slope	8.382e-005 to 0.0001224	−5.870e-005 to 1.061e-005	0.0001125 to 0.0002034
Goodness of Fit			
• Degrees of Freedom	169	169	168
• R square	0.3973	0.01098	0.2189
• Absolute Sum of Squares	0.001312	0.004239	0.007126
• Sy.x	0.002787	0.005008	0.006513
Number of points			
• Analyzed	171	171	171

**Table 5:** Filovirus Glycoprotein (GP 1, 2) host specific IgG among Negative Controls. This table represents host specific IgG levels among 9 negative controls

	Host Specific IgG		
	UG-Filo-Peptide 1	UG-Filo-Peptide 2	UG-Filo-Peptide 3
Best-fit values			
• YIntercept	0.4165	0.4743	−0.1944
• Slope	−0.04364	−0.02456	0.2225
Std. Error			
• YIntercept	0.2465	0.03052	0.2744
• Slope	0.04380	0.005423	0.04877
95% Confidence Intervals			
• YIntercept	0.1125 to 0.7205	0.4115 to 0.5372	−0.7596 to 0.3708
• Slope	−0.1339 to 0.04657	−0.03573 to − 0.01339	0.1220 to 0.3229
Goodness of Fit			
• Degrees of Freedom	25	25	25
• R square	0.03819	0.4507	0.4542
• Absolute Sum of Squares	8.635	0.1324	10.70
• Sy.x	0.5877	0.07276	0.6543
Number of points			
• Analyzed	27	27	27

**Table 6:** Filovirus Glycoprotein (GP 1, 2) host specific IgG among EVD survivor samples. This table paints a picture of host specific IgG levels among 92 gamma irradiated EVD survivor samples

	Host Specific IgG		
	UG-Filo-Peptide 1	UG-Filo-Peptide 2	UG-Filo-Peptide 3
Best-fit values			
• YIntercept	1.7181	1.8197	1.7197
• Slope	0.01042	0.02079	0.01126
Std. Error			
• YIntercept	0.08487	0.09550	0.07882
• Slope	0.001047	0.001192	0.0009768
95% Confidence Intervals			
• YIntercept	0.3010 to 3.1352	0.3268 to 3.3125	0.4145 to 3.0248
• Slope	0.008361 to 0.01248	0.01844 to 0.02314	0.009336 to 0.01318
Goodness of Fit			
• Degrees of Freedom	274	271	277
• R square	0.4655	0.5286	0.4242
• Absolute Sum of Squares	163.2	201.8	144.5
• Sy.x	0.7719	0.8629	0.7222
Number of points			
• Analyzed	276	276	276

**Table 7:** Summary of Statistical Characteristics of IgG ELISA Results among EVD survivors. This table summarizes statistical characteristics of IgG levels among EVD survivor samples

		PEPTIDE-1	PEPTIDE-2	PEPTIDE-3	TOTALS	AVERAGES
#	Statistical Function	Value(s)				
1	Sample size (N)	94	94	94	282	**94**
2	SUM (Σ) OD	65.54183297	69.40603	88.86964	223.8175	**74.60583**
3	Average OD	0.697253542	0.738362	0.955587	2.391203	**0.797068**
4	VAR	0.808399425	1.582093	0.720303	3.110795	**1.036932**
4	Σ (Deviance)^2^	76.34237	214.0516	71.75194	362.1459	**120.7153**
5	Σ (Deviance)^2^/(N-1)	0.820886	2.30163	0.771526	3.894042	**1.298014**
6	STD	0.906027	1.517112	0.878366	3.301505	**1.100502**
7	MAX	2.586364	2.397667	2.869666	7.853697	**2.617899**
8	MIN	−0.45733	−0.71833	−0.07133	−1.24699	**−0.41566**
9	RANGE	3.043698	3.116	2.941	9.100698	**3.033566**
10	MEDIAN	1.293182	1.178334	1.227666	3.699182	**1.233061**

**Table 8:** Filovirus Glycoprotein (GP 1, 2) antigen levels among EVD survivor samples. This table posts filovirus Glycoprotein (GP 1, 2) antigen levels among 33 EVD survivor samples and recombinant EBOV GP as positive control

	Filovirus GP1, 2 Antigen	
	PAb 1	PAb-2
Best-fit values		
• YIntercept	0.1108	0.2391
• Slope	−0.001581	−0.006704
Std. Error		
• YIntercept	0.008089	0.02180
• Slope	0.0004032	0.001087
95% Confidence Intervals		
• YIntercept	0.09479 to 0.1269	0.1958 to 0.2823
• Slope	−0.002381 to −0.0007811	−0.008860 to − 0.004548
Goodness of Fit		
• Degrees of Freedom	100	100
• R square	0.1333	0.2756
• Absolute Sum of Squares	0.1596	1.160
• Sy.x	0.03995	0.1077
Number of points		
• Analyzed	102	102

#### Antigen detection EIA

**Fig. 8:**
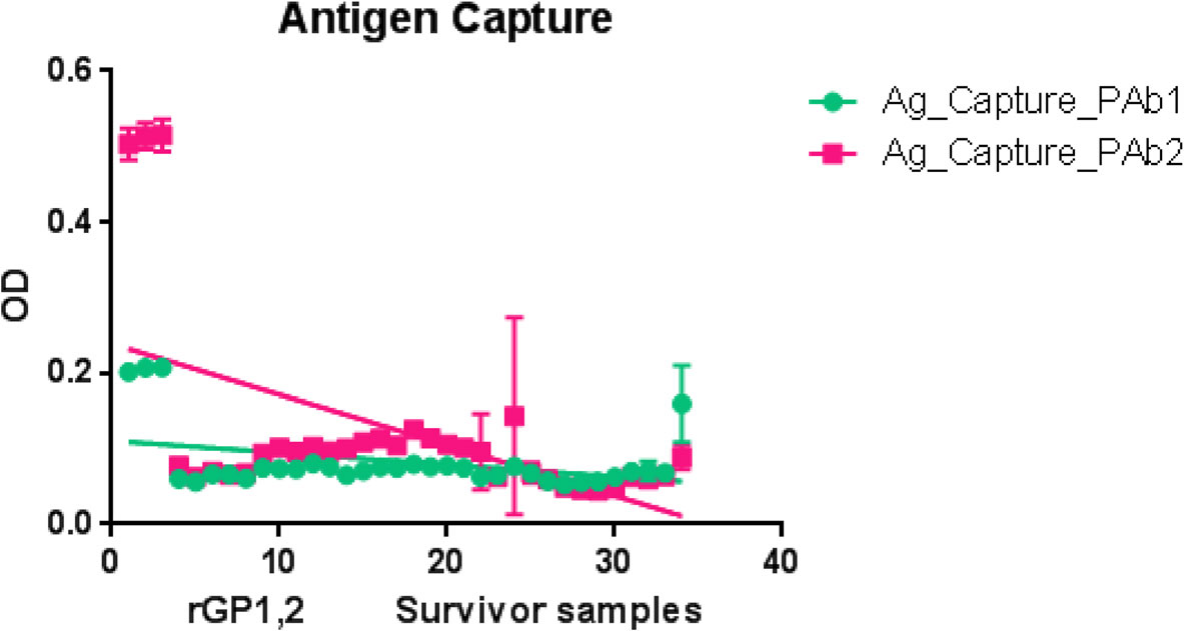
Filovirus GP1, 2 Antigen levels among EVD survivor serum samples. This figure shows levels of filovirus GP1, 2 antigens among the 33 survivor serum samples evalutated relative to the recombinant EBOV GP1, 2 pre-protein. No filovirus GP1, 2 was detected among survivor samples relative to the positive control

*Filovirus* Gp1, 2 antigen captured by two New Zealand derived polyclonal antibodies (PAb- A005345 and PAb-A005346: immunogens UG-Filo-Peptide 1 & UG-Filo-Peptide 3, respectively) was absent at ODs < 0.20 relative to recombinant protein positive controls at ODs = 0.50 (see Table 8 and Fig. 8). Overall, filovirus GP1, 2 Ag levels among all 33 survivor samples and 1 positive control (for 102 entries) detected by PAb- A005345 were 0.1108 (95% CI: 0.09479 to 0.1269) relative to 0.2391 (95% CI: 0.1958 to 0.2823) of PAb-A005346 (Table 8 and Fig. 8; alongside Additional file 3: S3); showing that PAb-A005346 had superior performance. Rowe AK, et al. (1999) found that all specimen obtained 3–6 days after symptoms began tested positive for antigen, and antigen positivity disappeared 7–16 days after symptoms began [38]. Since these were survivor serum samples collected after the convalescent stage of EVD, it is not surprising that all samples tested were antigen negative. Inquisitively, it remains unclear what the impact of gamma-irradiation is on the integrity of the target filoviral antigen, GP1, 2 pre-protein.

## Discussion

We present 3 conserved linear B cell epitopes of ebola virus GP1,2 preporotein that are validated in the present study as targets for the R & D of RDTs for EVD caused by all ebola virus species. One of the three epitopes exhibits *in-silico* conservation within all 4 *Ebolavirus* and 1 *Marburgvirus* species’ (*Maburg Marburg virus*, MARV) GP, promising duo-application to marburg-virus disease (MVD). Whereas 2 RDTs for the Zaire *ebolavirus* (EBOV) species have recently emerged on the market (Corgenix ReEBOV® and OraSure Technlogies., Inc. OraQuik® EBOV rapid antigen tests), it is unclear if they can detect other *Ebolavirus* species and or MARV [12, 13]. Within the equatorial African village setting, however, the need is for RDTs that can be used at the POC to rule out the causative agents of highly fatal VHFs—since many endemic illnesses like malaria, typhoid and Lassa fever present with a similar prodrome [1–38]. Overall, POC detection of filovirus related VHFs can enable early detection, response and control, especially since the available technologies for filovirus diagnosis lack the user friendliness for POC [8, 12, 13]. Building on the hypothesis that GP1,2 preprotein forms an alternative target to the VP40 antigen detected by existing RDTs for EBOV, we set out to identify conserved B cell epitopes of GP1,2 preprotein for future synthesis of pan-filovirus RDTs. The results obtained using 94 EVD (SUDV) survivor samples suggest that these epitopes and their derivative antibodies are promising for the R & D of a RDT for EVD caused by all 4 ebola virus species. More validation with MARV samples is needed to designate duo-usage.

First, the distribution of B cell eitopes was in general found to be even across the entire length of all Gp1, 2 analysed (see Fig. 1: Plates A, B, C, D and D). However, the highest occurence of species-specific B cell epitopes of *Ebolavirus* spp. and MARV GP1,2 was found to reside in the region between amino acids positioned 350 and 500 in all GP1,2 (Table 1). The list of the longest species-specific epitopes is shown in Table 2. By multple sequence alignments, the epitope 97/80_GAFFLYDRLAST is common to GP1, 2 of all 4 *Ebolavirus* and the 1 *Marburgvirus* species (MARV). Epitopes 39_YEAGEWAENCY & 500_CGLRQLANETTQALQLFLRATTELR are only unique to GP1, 2 of the 4 *Ebolavirus* species (see Fig. 2 Plates A). Theoretically, those RDTs devised using the first epitope UG-Filo-peptide 1 and its derivative antibodies (polyclonal-PAb or monoclonal-MAb) or biologics like aptamers would thus be relevant for detecting both *Ebolavirus* and *Marburgvirus* spp. at the POC; while UG-Peptide 2 and 3 RDTs would enable specific diagnosis of EVD. No mis-matches were found across the NCBI human, microbial, protozoal and viral databases for epitopes UG-Filo-Peptides 1 and 2. UG-Filo-Petide 3 was, *however,* homologous to GP2 of the *Lloviu cuevavirus* (Lloviu virus or simply LLOV) and an uncharacterized protein of the bacteria Provetella *sp. CAG.1124* (see Fig. 2: Plate B). Cuevavirus is a genus of the family *Filoviridae*. Prevotella *spp o*n the other hand, are gram negative bacteria of the oral and vaginal flora that cause anaerobic infections of the respiratory tract. These—particularly the latter, might in part explain two IgM outlier hits obtained among negative controls with UG-peptide 3 (Fig. 7a). All the 3 epitopes were present in the GP1, 2 of the sequences of the Zaire ebolavirus (EBOV) responsible for the 2013-to-2016 West African EVD outbreak (see Fig. 2: Plate C) [34]. Lastly, each of the 3 epitopes mapped on 3 dimensional crystal structure of EBOV GP1, 2 as shown in Fig. 3: Plates A, B and C) [10]. These and other associated data are described in the World Intellectual Organization (WIPO) patent application # PCT/IB2014/066251 [32]. Synthetic analogues of the epitopes UG-Filo-Peptide 1, 2 and 3(Fig. 4: Plates A, B, C, D, E and F), recombinant EBOV GP1, 2 cloned and expressed in HEK293-6E cell-lines (Fig. 5: Plates A and B; plus Additional file 1: S1) alongside the derivative polyclonal antibodies of UG-Filo-Peptide 1 and 3(PAbs- A005345 and A005346 respectively; see Additional file 2: S2) were used for antibody and antigen enzyme immune-assays (EIAs) with survivor serum samples (Additional file 3: S3).

Second, (a) filovirus Gp1, 2 host specific IgM levels were found to similarly be low (ODs < 0.04; 95% CI: 0.02837 to 0.04033) among 9 negative controls (see Table 3 and Fig. 6: Plate A) and 57 survivor samples analyzed (see Table 4 and Fig. 6: Plate B). The two outliers hits for IgM within negative controls picked by UG-peptide 3 (Fig. 7 A) are more likely to be due to either cross reactivity with another pathogen (see Fig. 2, plate B), or higher affinity, rather than the possibility that these were infected samples since they were drawn from a non-VHF endemic setting. This is in-line with the immunopathogenesis of EVD—wherein IgM appears between 2 and 9 days after symptom onset, and disappears between 30 and 168 days after onset [34–38]. Thus, while no IgM was presently detected (and is indeed expected to be) present within the survivor serum used in this validation, those RDTs that detect host specific IgM are relevant towards detecting acute *Ebolavirus* spp. and or MARV infections. (b) Consistent with the immuno-biology of EVD, host specific IgG levels were elevated (av. ODs > 1.7525 [95% CI: 0.3010 to 3.1352]) among the 92 survivor samples relative to 9 negative controls (av. ODs < 0.2.321 [95% CI: -0.7596 to 0.5372]) (see Tables 5 and 6, alongside Fig. 7 Plates A and B, respectively). All 3 epitopes performed well for purposes of capturing host-specific IgG across 94 survivor serum. However, UG-peptide 3 had relatively higher specificity relative to the other two peptides across the statistical parameters used (see Table 7 and Fig. 7 Plate C). These data are consistent with prior studies that found that IgG antibody appears between 6 and 18 days after symptom onset and persists for life [35–40]. It is therefore not suprising that IgG was abundant across the survivor serum samples used, as early reported [35–40]. Those RDTs detecting host IgG responses, would be relevant for clinical and epidemiological follow-up. Lastly, (c) filovirus Gp1, 2 antigen was absent at ODs < 0.20 relative to recombinant protein positive controls at ODs = 0.50 (see Table 8 and Fig. 8). Details of GP1, 2 antigen and antibody detection are available in Additional file 3: S3. Rowe AK, et al. (1999) previously reported that antigen positivity disappeared 7–16 days after symptoms began [39]. Because survivor samples were collected after the convalescent stage of EVD, it is therefore not surprising that all samples tested were antigen negative. In prospect, however, those RDTs based on Ag detection should offer a confirmatory test for acute infection with *Ebolavirus* spp. and or MARV. The impact of gamma-irradiation on the integrity of the target filoviral antigen, GP1, 2 pre-protein is unknown [8, 12, 13, 40].

Several challenges remain to be tackled before these tests usable in the clinic or at POCs. *First*, the localization of the target epitopes on native structure GP may be concealed to detection by virue of intracellular localization, mannose-glycosylations, or disulfide molecularization. In this respect, it is not only important to target the extra-cellular domain of GP, but pre-treatment of native GP in sample with glycosidases to remove mannose-glycans, endopeptidases to denature the 3-D structure of GP, and reducing agents to break the disulfide bond might enhance capture. Fortunately, the 3 epitopes are mapped to the ssGP, GP1 and GP2 components of the 3-D cristal structure of EBOV-GP in conjunction with human antibody(see Figs. 7 and 8) [10]. *Second*, RDTs targeting GP carry the risk of yielding false positives as a result of confounding among persons who will have been vaccinated with some of the prospective trial vaccines against *Ebolavirus* spp. and MARV based on viral vectors that actively express filovirus GP, and possibly attenuated forms of the virus [9, 11]. This brings to fore a need to incorporate a protein water-marker to distinguish vaccine-expresed GP from infectious filovirus GP. *Third*, the fact that we used negative controls from a non-filovirus setting might demand more optimization of what a true negative or positive result is using samples from endemic seetings. *Fourth,* more validation with MARV samples is needed to confirm the usability towards R & D of pan-filovirus RDTs. *Last but not least,* that UG-Filo-Peptide 3 exhibits *in-silico* homology to Cuevavirus and Prevotella *spp*, demands that secondary testing to rule out false positive issuing from acute infection with for Lloviu (LLOV) and or Provetella *spp* be conducted at reference laboratories.

In conclusion, these conserved B cell epitopes of filovirus Gp1, 2 and their derivative antibodies (or biologics like aptamers) are targets presently validated for R & D of RDTs for testing for EVD at the POC. More validation studies with MARV samples are needed to designate duo or pan-filovirus usage. Overall, RDT prototypes that detect filovirus GP Ag and or its host-specific IgM are usable towarded detecting acute filovirus infections, while those prototypes detecting host specific IgG can be applied for survivor studies and or monitoring for vaccine efficiency. Supplementary optimization is still needed to bring these tests to the POC.

## Accession numbers

The swiss prot accession #s for the 4 *Ebolavirus* spp. (*Zaire ebolavirus, Tai Forest ebolavirus, Sudan ebolavirus,* and *reston ebolavirus*) and 1 *Marburgvirus* (*MARV*) species are respectively >sp.|P87671|; >sp.|Q66810|; >sp.|Q66798|; >sp.|Q91DD8| and > sp.|Q1PD50|. The PDB entry for EBOV Gp used is 3CSY.

## Additional files

Additional file 1: S1 Cloning, Expression and Purification of EBOV GP1, 2 in HEK293-6E mammalian cell-lines. This file details the cloning, expression and purification of EBOV GP1, 2 in HEK293-6E mammalian cell-lines. (PDF 1555 kb)

Additional file 2: S2 Certificate of Analysis of New Zealnad Rabbit-derived Polyclonal Antibodies. This file shows the certificate of polyclonal antibodies of UG-Filo-Peptide 1 and 3(PAbs- A005345 and A005346 respectively) generated within New Zealand rabbits. Note the ELISA titer of > 1:128. (PDF 109 kb)

Additional file 3: S3 Details of survivor serum samples alongside results of Antigen and Antibody detection EIAs. This file describes the survivor serum samples alongside results of Antigen and Antibody detection EIAs. (XLSX 95 kb)

## Abbreviations

AG: Antigen
BDBV: Bundibugyo ebolavirus
BSA: Bovine serum albumin
CDC: Centres for Disease Control, Atlanta
EBOV: Zaire ebolavirus
EDCTP: Europena Developing Country Clinical Trials Partnership
EIA: Enzyme immuno-adsorbent assays
EVD: Ebola virus disease
GCC: Grand Challenges Canada
GP: Glycoprotein
IgM: Immunoglogulin M
IgGP: Immunoglobulin G
MARV: Marburg Marburgvirus
PBS: Phosphate buffered saline
PCR: Polymerase Chain Reaction
POC: Point of care
R & D: Research and Development
RDT: Rapid Diagnostic Test(s)
RESTV: Reston ebolavirus
SUDV: Sudan ebolavirus
TAFV: Tai Forest ebolavirus
VHF: Viral hemorragic fevers
WHO: World Health Organization
