# Supplementary material for: Identity and validity of conserved B cell epitopes of filovirus glycoprotein: towards rapid diagnostic testing for Ebola and possibly Marburg virus disease
Source: BMC Infect Dis. 2018 Oct 3;18:498. doi: 10.1186/s12879-018-3409-x (PMC6171133; doi:10.1186/s12879-018-3409-x)

FINAL REPORT

CONFIDENTIAL

Transient Expression and Purification of Recombinant GP

Project Order # 340397-1

Date: 01/27/2014

This report is provided by:

GenScript USA Inc
860 Centennial Ave
Piscataway, NJ 08854
United States of America

This study was conducted according to the procedures described in this report. All data presented are authentic, accurate and correct to the best of our knowledge. Information and materials provided by this investigation are for ***research use*** only.

CONFIDENTIAL

SIGNATURE

This report is being submitted by the following personnel.

Prepared by

Litiy
Staff, Bioprocess
GenScript USA Inc

Andy Wang
Production Manager, Bioprocess
GenScript USA Inc

Approved by

Zhen-Yu Liu, Ph.D.
Director, Bioprocess
GenScript USA Inc

Tim Xia, Ph.D.
VP, Biological Services
GenScript USA Inc

CONFIDENTIAL

Index

1. Project Summary 4
2. Experimental Methods 4
2.1 Cell Culture and Transient Transfection 4
2.2 Purification and Analysis 4
3. Experimental Results 4
3.1 GP purified from cell culture supernatant 4
3.2 GP purified from cell lysate 5
4. Conclusion and Discussion 7
5. Packing List of Order 340397-1 7
6. Product Information 9
Appendix 10

CONFIDENTIAL

1. Project Summary

This report describes the transient expression of GP protein in suspension 293-6E cells using serum free medium, followed by one-step purification from both cell culture supernatants and total membrane proteins extracted from cell lysate.

2. Experimental Methods

2.1 Cell Culture and Transient Transfection

293-6E cells were grown in serum-free FreeStyle™ 293 Expression Medium (Life Technologies, Carlsbad, CA, USA). The cells were maintained in Erlenmeyer Flasks (Corning Inc., Acton, MA) at 37°C with 5% CO_2 on an orbital shaker (VWR Scientific, Chester, PA). One day before transfection, the cells were seeded at an appropriate density in Corning Erlenmeyer Flasks. On the day of transfection, DNA and PEI (Polysciences, Eppelheim, Germany) were mixed at an optimal ratio and then added into the flask with cells ready for transfection. Cell pellets and cell culture supernatant were collected at day 4 post-transfection for further purification.

2.2 Purification and Analysis

Cell culture supernatant and total membrane proteins extracted from cell lysate were loaded onto Ni Sepharose 6 FF 0.3 mL resin (GE Healthcare, Cat.No.17-5318-02), respectively. After washing and elution, the purified protein was analyzed by SDS-PAGE and Western blot by using standard protocols for molecular weight and purity.

3. Experimental Results

3.1 GP purified from cell culture supernatant

The plasmid DNA encoding GP was transiently transfected into 100 mL suspension 293-6E cells. Cell supernatants were collected at day 4 post-transfection and the target protein was purified by loading onto Ni Sepharose 6 FF 0.3 mL resin. The samples from purification step were analyzed by SDS-PAGE and Western blot analysis as shown in Figure 1. The

CONFIDENTIAL

primary antibody for Western blot was Mouse-anti-his mAb (GenScript, Cat.No.A00186).

A. SDS-PAGE **B. Western blot**

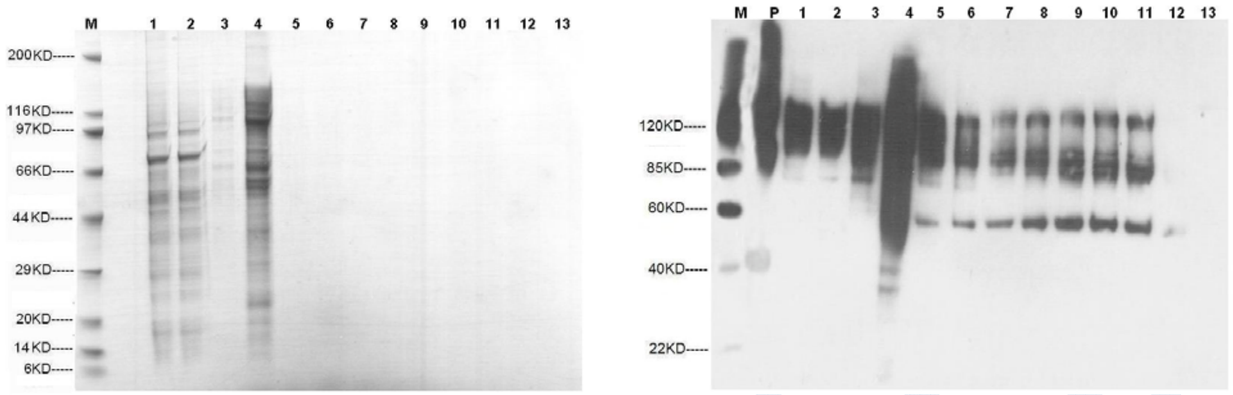

Figure 1. SDS-PAGE and Western blot analysis of day 4 cell culture supernatant after one-step purification

Lane M: Marker
Lane 1: Cell culture supernatant after centrifugation
Lane 2: Flow-through
Lane 3~4: 50mM imidazole eluted fractions
Lane 5~7: 100mM imidazole eluted fractions
Lane 8~11: 250mM imidazole eluted fractions
Lane 12~13: 1M imidazole eluted fractions
Lane P: Multiple-tag (GenScript, Cat.No.M0101) as positive control

We have tried to purify the target protein GP from cell culture supernatant. However, very little protein could be detected in eluted fractions. It indicated that the protein was positively expressed in suspension 293-6E cells. However, the target protein was not bound well onto the resin.

3.2 GP purified from cell lysate

Cell lysate collected from day 1 to day 4 post-transfection were analyzed by SDS-PAGE and Western blot as shown in Figure 2. The cells were harvested at day 4 post-transfection, lysed to collect target protein. The extracted membrane proteins were loaded onto Ni Sepharose 6 FF 0.3 mL resin to capture target protein, and analyzed by SDS-PAGE and Western blot as shown in Figure 3 ~ Figure 4. The primary antibody for Western blot was Mouse-anti-his mAb (GenScript, Cat.No.A00186).

A. SDS-PAGE B. Western blot

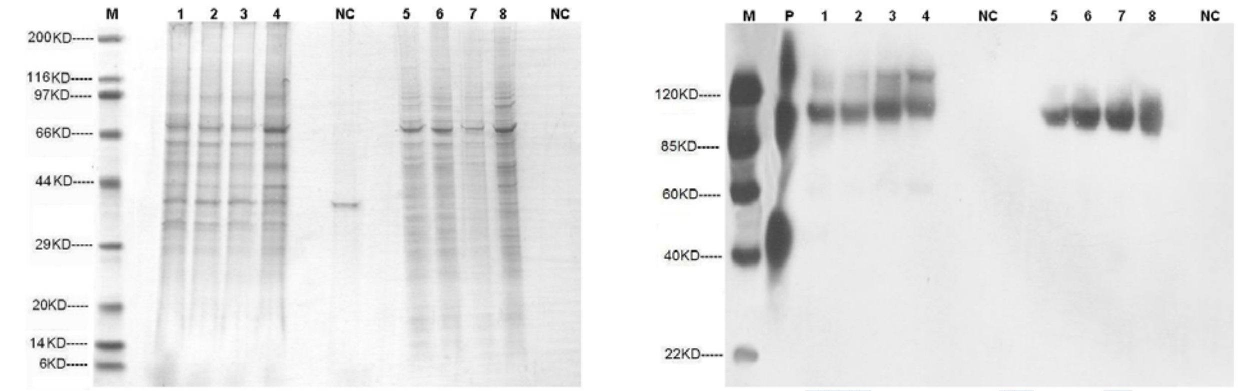

Figure 2. SDS-PAGE and Western blot analysis of cell lysate from day 1 to day 4 post-transfection

Lane M: Marker
Lane 1~4: Cell lysate from day 1 to day4 post-transfection with RIPA buffer
Lane 5~8: Cell lysate from day 1 to day4 post-transfection with hypotonic buffer
Lane NC: Negative control
Lane P: Multiple-tag (GenScript, Cat.No.M0101) as positive control

A. SDS-PAGE B. Western blot

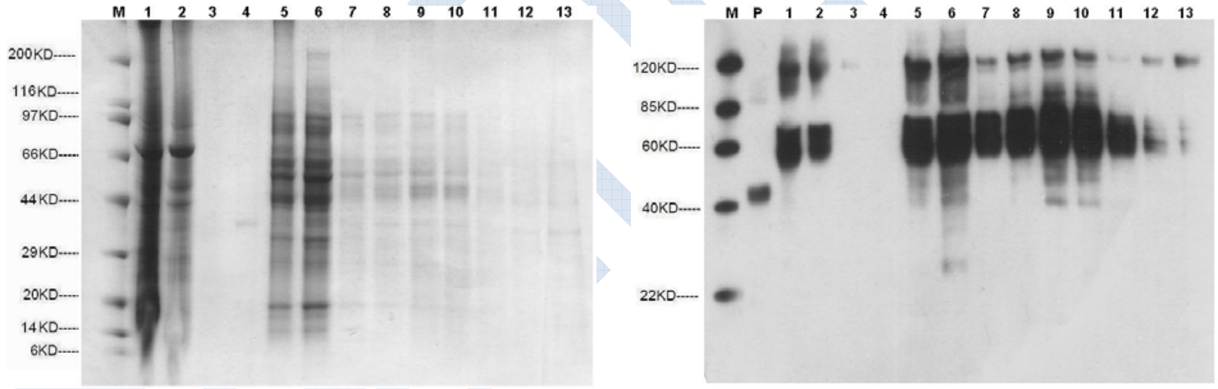

Figure 3. SDS-PAGE and Western blot analysis of GP from day 4 cell lysate after one-step purification

Lane M: Marker
Lane 1: Cell lysate supernatant
Lane 2: Flow-through
Lane 3~4: 50mM imidazole eluted fractions
Lane 5~8: 100mM imidazole eluted fractions
Lane 9~12: 250mM imidazole eluted fractions
Lane 13: 1M imidazole eluted fraction
Lane P: Multiple-tag (GenScript, Cat.No.M0101) as positive control

The fractions containing purified GP were collected and desalted to PBS, with 0.1% DDM,

pH 7.2. The final products were analyzed by SDS-PAGE and Western blot analysis as shown in Figure 4. The primary antibody for Western blot was Mouse-anti-his mAb (GenScript, Cat.No.A00186).

A. SDS-PAGE **B. Western blot**

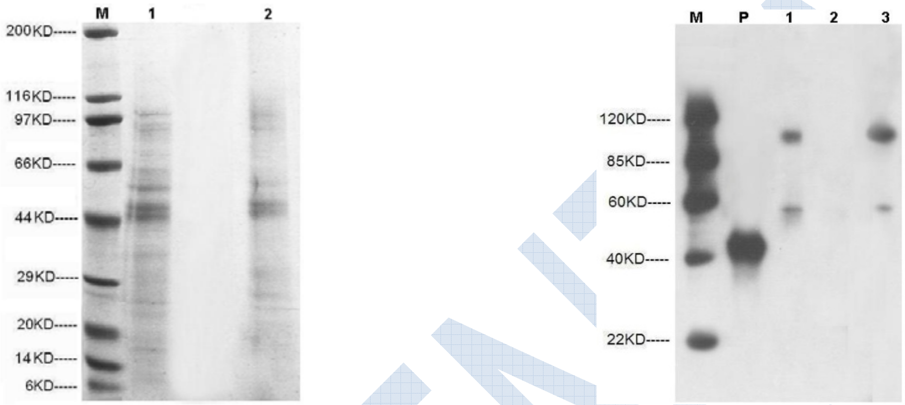

Figure 4. SDS-PAGE and Western blot analysis of purified GP from cell lysate after desalting

Lane M: Protein Marker
Lane 1: Reducing conditions
Lane 2: Non-reducing conditions
Lane P: Multiple-tag (GenScript, Cat.No.M0101) as positive control

4. Conclusion and Discussion

We have successfully expressed and purified GP in 293-6E cells from cell lysate. The purified protein was detected with estimated molecular weight of ~100 kDa (Cal.M.W. ~71.67kDa) on Western blot. 0.02 mg GP (Concentration: 0.2 mg/mL, Purity: ~50%) were delivered.

5. Packing List of Order 340397-1

Protein from cell lysate

(Shipping condition: -196℃)

- Name: GP
- Concentration: 0.2 mg/mL
- Volume per vial: 0.1 mL
- Purity: ~50%

CONFIDENTIAL

- Buffer: PBS, with 0.1% DDM, pH 7.2
- Lot No.: 340397S1/B0491401
- Total protein: 0.02 mg
- Number of vials: 1
- Store at -80℃

CONFIDENTIAL

6. Product Information

**Order Number:**	340397-1
**Protein Name:**	GP
**Fusion Tag(s):**	N-His6
**Lot Number:**	340397S1/B0491401

**Codon Optimization:**	Yes
**Expression System & Vector:**	293-6E cells, Mammalian Expression Vector pTT5
**Expression Optimization:**	Yes
**Expression Scale:**	100 mL
**Tag Removal:**	No
**Purification:**	Protein was obtained from cell lysate, followed by one step purification by Ni Sepharose 6 FF resin

**Package:**	0.02 mg, 0.1 mL/vial, 1 vial
**Concentration:**	0.2 mg/mL as determined by Bradford assay
**Purity:**	About 50% as estimated by densitometric analysis of the Coomassie Blue-stained SDS-PAGE gel
**Storage and Handling:**	Store at -80°C. Aliquots should be stored at the same temperature after first use to avoid multiple freeze-thaws
**Storage Buffer:**	PBS with 0.1% DDM, pH 7.2

CONFIDENTIAL

GenScript
Make Research Easy

One Stop Solution for Biology Research

Appendix

Cloning Strategy:

EcoRI -- Kozak sequence -- ATG -- Artificial Signal Peptide -- 6xHis -- Target Protein Envelope glycoprotein (33-671 aa optimized for HEK293) -- stop codon -- HindIII

DNA Sequence:

2034 bp

```
GAATTCccGCCGCCACCATGGGCTGGTCCTGTATTATCCTGTTTCTGGTCGCAACCGCTACTGGCGTCCACTCACATCA
TCATCATCATCACATGCCTCTGGGAGTGGTCACTAATAGCACCCTGGAAGTGACCGAGATCGATCAGCTGGTCTGTAAGG
ACCACCTGGCTTCTACAGATCAGCTGAAAAGTGTGGGCCTGAACCTGGAGGGATCAGGCGTCAGCACCGATATCCCTTCT
GCAACAAAGCGATGGGGCTTCCGGAGTGGAGTGCCACCTAAAGTGGTCTCATACGAAGCCGGCGAGTGGGCTGAAAACTG
CTATAATCTGGAGATCAAGAAACCTGATGGGAGCGAATGTCTGCCACCACCTCCAGACGGAGTGAGGGGATTCCCAAGAT
GCAGATACGTCCACAAGGCTCAGGGGACCGGACCTTGTCCAGGGGACTATGCCTTTCATAAAGATGGAGCTTTCTTTCTG
TACGACCGGCTGGCCTCTACCGTGATCTATAGAGGAGTCAATTTCGCTGAGGGCGTGATCGCATTTCTGATTCTGGCCAA
GCCAAAAGAAACCTTCCTGCAGAGCCCCCCTATTCGCGAGGCCGTGAATTACACAGAAAACACTAGCTCCTACTATGCTA
CAAGCTACCTGGAGTATGAAATCGAGAACTTTGGCGCCCAGCACTCCACCACACTGTTCAAGATCGATAACAACACATTT
GTGCGGCTGGACAGACCCCATACTCCTCAGTTCCTGTTTCAGCTGAATGACACTATCCACCTGCATCAGCAGCTGAGCAA
CACTACCGGGCGGCTGATTTGGACACTGGACGCAAACATCAATGCCGATATTGGAGAGTGGGCATTCTGGGAAAACAAGA
AAAATCTGTCCGAGCAGCTGAGAGGGGAGGAACTGAGCTTTGAAGCCCTGTCCCTGAATGAAACCGAGGACGATGACGCC
GCTTCTAGTAGAATCACCAAGGGAAGGATTTCCGATAGGGCCACACGCAAGTACTCAGACCTGGTGCCAAAAAACAGCCC
CGGAATGGTCCCTCTGCACATCCCAGAAGGCGAGACAACTCTGCCATCCCAGAACAGCACTGAGGGACGGAGAGTGGGCG
TCAACACCCAGGAGACAATTACTGAAACCGCAGCCACCATCATTGGGACAAACGGAAATCACATGCAGATCTCTACCATC
GGCATTCGGCCCTCAAGCTCCCAGATTCCCTCTAGTTCACCTACCACAGCTCCCAGTCCTGAGGCACAGACACCAACTAC
CCATACTAGTGGGCCCTCAGTGATGGCCACCGAGGAACCCACAACTCCACCAGGAAGCTCCCCAGGACCAACCACAGAGG
CACCTACACTGACTACCCCAGAAAATATCACAACTGCCGTGAAGACTGTCCTGCCTCAGGAGTCTACCAGTAACGGCCTG
ATCACAAGCACTGTGACCGGCATTCTGGGGTCCCTGGGACTGCGCAAACGATCTAGGCGCCAGACAAATACTAAGGCTAC
AGGAAAATGCAACCCCAATCTGCACTACTGGACTGCACAGGAGCAGCATAACGCTGCAGGCATCGCTTGGATTCCATACT
TCGGCCCCGGGGCAGAGGGCATCTATACCGAAGGGCTGATGCATAACCAGAATGCACTGGTGTGCGGACTGCGACAGCTG
GCAAATGAGACCACACAGGCCCTGCAGCTGTTCCTGCGCGCCACTACCGAACTGCGAACCTATACAATCCTGAACAGGAA
GGCTATTGATTTTCTGCTGCGACGGTGGGGAGGAACCTGCCGCATCCTGGGACCAGACTGCTGTATTGAGCCTCACGATT
GGACTAAGAATATCACCGACAAAATTAACCAGATCATTCATGACTTCATCGACAACCCTCTGCCAAACCAGGACAATGAT
GACAACTGGTGGACCGGCTGGAGACAGTGGATCCCTGCCGGGATTGGCATCACTGGGATTATTATCGCCATCATCGCCCT
GCTGTGCGTCTGTAAACTGCTGTGCTGATAAGCTT
```

Protein Sequence:

669 aa

```
MGWSCIILFLVATATGVHShhhhhhmplgvvtnstlevteidqlvckdhlastdqlksvglnlegsgvstdipsatkrwg
frsgvppkvvsyeagewaencynleikkpdgseclppppdgvrgfprcryvhkaqgtgpcpgdyafhkdgafflydrlas
tviyrgvnfaegviaflilakpketflqsppireavnytentssyyatsyleyeienfgaqhsttlfkidnntfvrldrp
htpqflfqlndtihlhqqlsnttgrliwtldaninadigewafwenkknlseqlrgeelsfealslnetedddaassrit
kgrisdratrkysdlvpknspgmvplhipegettlpsqnstegrrvgvntqetitetaatiigtngnhmqistigirpss
sqipssspttapspeaqtptthtsgpsvmateepttppgsspgptteaptlttpenittavktvlpqestsnglitstvt
gilgslglrkrsrrqtntkatgkcnpnlhywtaqeqhnaagiawipyfgpgaegiyteglmhnqnalvcglrqlanettq
alqlflrattelrtytilnrkaidfllrrwggtcrilgpdcciephdwtknitdkinqiihdfidnplpnqdnddnwwtg
wrqwipagigitgiiiaiiallcvckllc
```

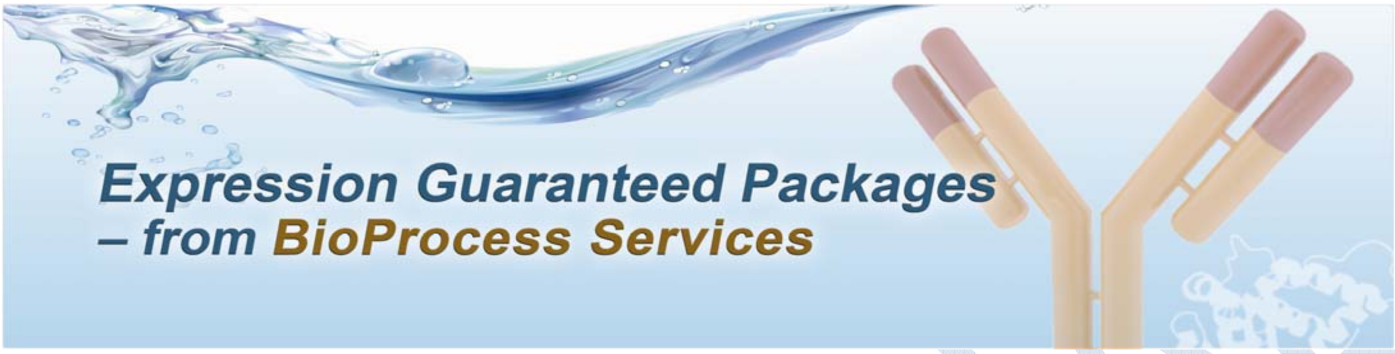

Guaranteed antibody expression in mammalian cells

MamPower™ Guaranteed Antibody Package (SC1259A) Promotion
Guaranteed expression of recombinant antibodies in mammalian cells

- 50 mg antibody Guaranteed – or it's FREE*
- Starting from $8,499 (Gene synthesis fee included)

For more details, please visit http://www.genscript.com/bioprocess_promotion_a.html

Guaranteed 3mg Protein – or it is FREE

MamPower™ Guaranteed Protein Package (SC1259P) Promotion
An unprecedented guarantee package that no others can offer

- 3 mg protein Guaranteed
- Starting from $8,900 (Gene synthesis fee included)

For more details, please visit http://www.genscript.com/bioprocess_promotion_p.html

*Certain rules may apply, please check our website for details

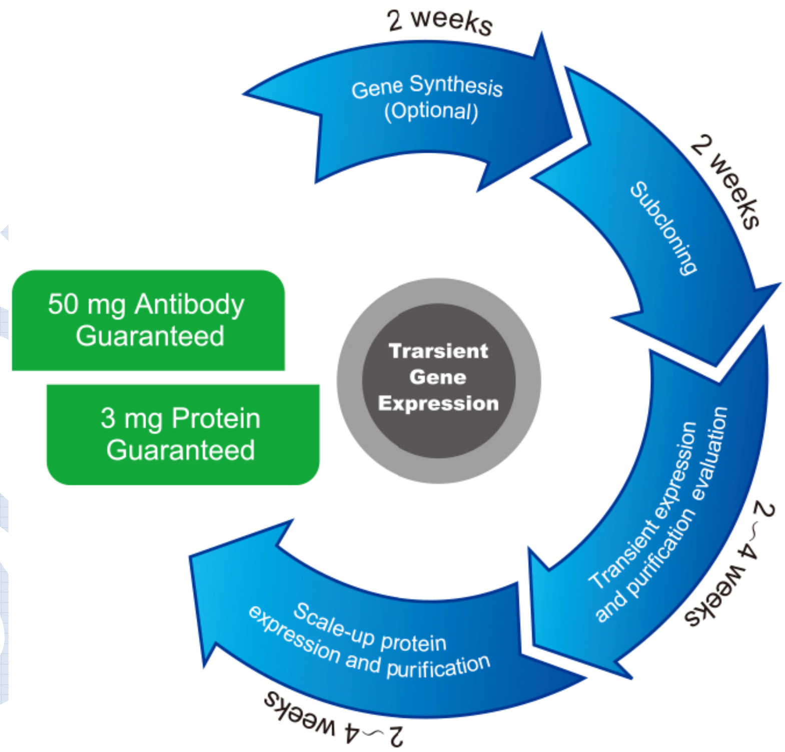

Supplement: Supplementary file 1 — S1 Cloning, Expression and Purification of EBOV GP1, 2 in HEK293-6E mammalian cell-lines. This file details the cloning, expression and purification of EBOV GP1, 2 in HEK293-6E mammalian cell-lines. (PDF 1555 kb) [file 12879_2018_3409_MOESM1_ESM.pdf]
