# Supplementary material for: Identity and validity of conserved B cell epitopes of filovirus glycoprotein: towards rapid diagnostic testing for Ebola and possibly Marburg virus disease
Source: BMC Infect Dis. 2018 Oct 3;18:498. doi: 10.1186/s12879-018-3409-x (PMC6171133; doi:10.1186/s12879-018-3409-x)
Supplement: Supplementary file 2 — S2 Certificate of Analysis of New Zealnad Rabbit-derived Polyclonal Antibodies. This file shows the certificate of polyclonal antibodies of UG-Filo-Peptide 1 and 3(PAbs- A005345 and A005346 respectively) generated within New Zealand rabbits. Note the ELISA titer of > 1:128. (PDF 109 kb) [file 12879_2018_3409_MOESM2_ESM.pdf]

Custom Services for Research

CERTIFICATE OF ANALYSIS

1. Project Data:

**Date:**	**2014-5-15**			
**Order ID**	**AB130017**			
**Product ID:**	**A005345**		**A005346**	
**Antibody Name:**	**ARZ-ssGP**		**ARZ-GP2**	
**Antigen**	**YEAGEWAENCY**		**CGLRQLANETTQALQLFLRATTELR**	
**Peptide ID:**	**384921**		**384922**	
**Lot # of**	**A140123-QL2390**		**A140123-QL2391**	
**Concentration :**	**0.5mg/ml(HRP labelled)**		**0.5mg/ml(HRP labelled)**	
**Volumn :**	**6ml(before lyophilized)**		**6ml(before lyophilized)**	
**Animal ID :**	**RB4933**	**RB4934**	**RB4935**	**RB4936**
**Concentration :**	**1.05mg/ml**	**0.89mg/ml**	**0.79mg/ml**	**0.82mg/ml**
**Volumn :**	**3ml(before lyophilized)**	**3ml(before lyophilized)**	**3.5ml(before lyophilized)**	**3.5ml(before lyophilized)**
**Store Condition:**	**With 0.02% NaN3 before lyophilized. HRP labelled antibody doesn't contain the NaN3, and it is recommended to disolve to 1mg/ml with 1 :5000-1 :10000 woking dilution.**<br>**Store at 4℃ if to be used in less than 1 week after dissolved, for long-term storage at -20℃.**<br>**Antibody at working concentration shall be made and used in the same day.**			

2. Identification Data:

ELISA:

Coating Antigens: recombinant protein
Coating Concentration: 2 ug/ml, 100ul / well
Coating Buffer: Phosphate Buffered Saline, pH7.4
Secondary Antibody: Goat Anti-Rabbit IgG (H+L), HRP Conjugated
Elisa result for Antigen Affinity Purified Pab

GeneCust
Custom Services for Research

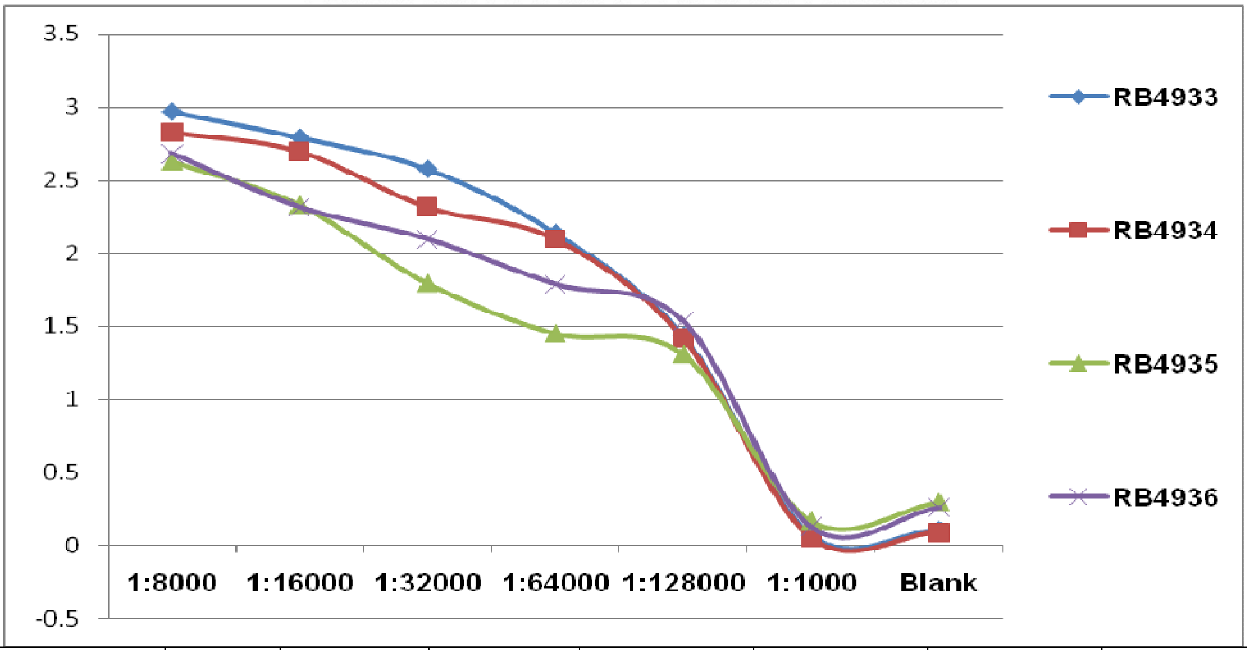

Animal ID	Blank	(-) 1 :1K	SA 1:8K	SA 1:16K	SA 1 :32K	SA 1 :64K	SA 1 :128K
RB4933	0.104	0.067	2.965	2.789	2.573	2.135	1.434
RB4934	0.091	0.054	2.829	2.698	2.316	2.098	1.423
RB4935	0.295	0.166	2.628	2.33	1.793	1.449	1.308
RB4936	0.262	0.131	2.684	2.316	2.098	1.789	1.538

(-) :Negative serum 1:1000 OD <0.2
SA: Peptide affinity purified Pab 1 :128K OD>1.0
Elisa Titer>1 :128K

Delivery list:

Product ID	Animal ID	Pre-Bleed(-)	Post-Bleed(+)	Peptide	Purified Antibody
A005345	RB4933	0.5-0.6ml/1 vial	0.5-0.6ml/1 vial	3mg/1 vial	3ml/1 vial
	RB4934	0.5-0.6ml/1 vial	0.5-0.6ml/1 vial		3ml/1 vial
HRP labelled A005345	——	——	——	——	6ml/1 vial
A005346	RB4935	0.5-0.6ml/1 vial	0.5-0.6ml/1 vial	3mg/1 vial	3.5ml/1 vial
	RB4936	0.5-0.6ml/1 vial	0.5-0.6ml/1 vial		3.5ml/1 vial
HRP labelled A005345	——	——	——	——	6ml/1 vial

Antibody use/dissolution

1.Add deionized or distilled water to reconstitute the lyophilized products and mix gently by inverting 5-6 times at room temperature.
2.It may take a few minutes to reconstitute a lyophilized product. If insoluble matter is observed, remove precipitation by centrifugation at 3000 rpm or higher for 10-15 min, transfer supernatant to a fresh tube.

3.For lyophilized antisera or preimmune sera, reconstitute to the original volume as described on the data sheet or report sheet.
4.For lyophilized purified antibodies, reconstitute to a final antibody concentration according to your applications or the recommended concentration on the data sheet.
5.The reconstituted antibody can be stored for 2-3 weeks at 2-8°C or for up to 12 months at -20°C or below. Avoid repeated freeze and thaw cycles.
6.Lyophilized product remains stable for at least one year if stored at -20°C or below.

Note: 0.02% of sodium azide was added as preservative before lyophilization.
